# Proteomic characterization of esophageal squamous cell carcinoma response to immunotherapy reveals potential therapeutic strategy and predictive biomarkers

**DOI:** 10.1186/s13045-024-01534-9

**Published:** 2024-03-15

**Authors:** Fahan Ma, Yan Li, Chan Xiang, Bing Wang, Jie Lv, Jinzhi Wei, Zhaoyu Qin, Yan Pu, Kai Li, Haohua Teng, Subei Tan, Jinwen Feng, Zhanxian Shang, Yunzhi Wang, Sha Tian, Changsheng Du, Yuchen Han, Chen Ding

**Affiliations:** 1 State Key Laboratory of Genetic Engineering and Collaborative Innovation Center for Genetics and Development, School of Life Sciences, Institutes of Biomedical Sciences, Human Phenome Institute, Zhongshan Hospital, Fudan University, Shanghai, 200433 China; 2 Department of Pathology, Shanghai Chest Hospital, Shanghai Jiao Tong University School of Medicine, Shanghai, 200030 China; 3 Key Laboratory of Spine and Spinal Cord Injury Repair and Regeneration of Ministry of Education, Orthopaedic Department of Tongji Hospital, School of Life Sciences and Technology, Tongji University, Shanghai, 200092 China

**Keywords:** Esophageal squamous cell carcinoma, Proteomics, Anti-PD1 immunotherapy, Platelets activation, Predictive markers, Immunotherapy response prediction

## Abstract

Immunotherapy is the first-line therapy for esophageal squamous cell carcinoma (ESCC), yet many patients do not respond due to drug resistance and the lack of reliable predictive markers. We collected 73 ESCC patients (including discovery cohort and validation cohort) without immune thrombocytopenia and undergoing anti-PD1 immunotherapy. Proteomic and phosphoproteomic analysis of 73 ESCC treatment-naive samples by mass spectrometry-based label-free quantification were applied to explore the potential resistant and sensitive mechanisms, and identify predictive markers of ESCC immunotherapy. Comparative analysis found the pathways related to immune and mitochondrial functions were associated with ESCC immunotherapy sensitivity; while platelet activation bioprocess showed negative correlation with CD8+ T cells and related to ESCC immunotherapy non-sensitivity. Finally, we identified 10 ESCC immunotherapy predictive biomarkers with high accuracy (≥ 0.90) to predict the immunotherapeutic response, which was validated in the independent cohort.

**Supplementary Information:**

The online version contains supplementary material available at 10.1186/s13045-024-01534-9.

**To the editor**

Immunotherapy has been the first-line therapy for ESCC, however, the object response rate (ORR) was only 54.2% [1–4]. Screening patients suitable for immunotherapy is challenging due to the limitation in the specificity and sensitivity of existing companion diagnostic markers, such as PD-L1 expression [5, 6].

We conducted comprehensive proteomic profiling of tumor biopsy derived from 73 immunotherapy treatment-naïve ESCC patients, including discovery cohort (53 patients) and validation cohort (20 patients) (Fig. 1A and Additional file 1: Table S1). A detailed description of materials and methods can be found in Additional file 1.

**Fig. 1:**
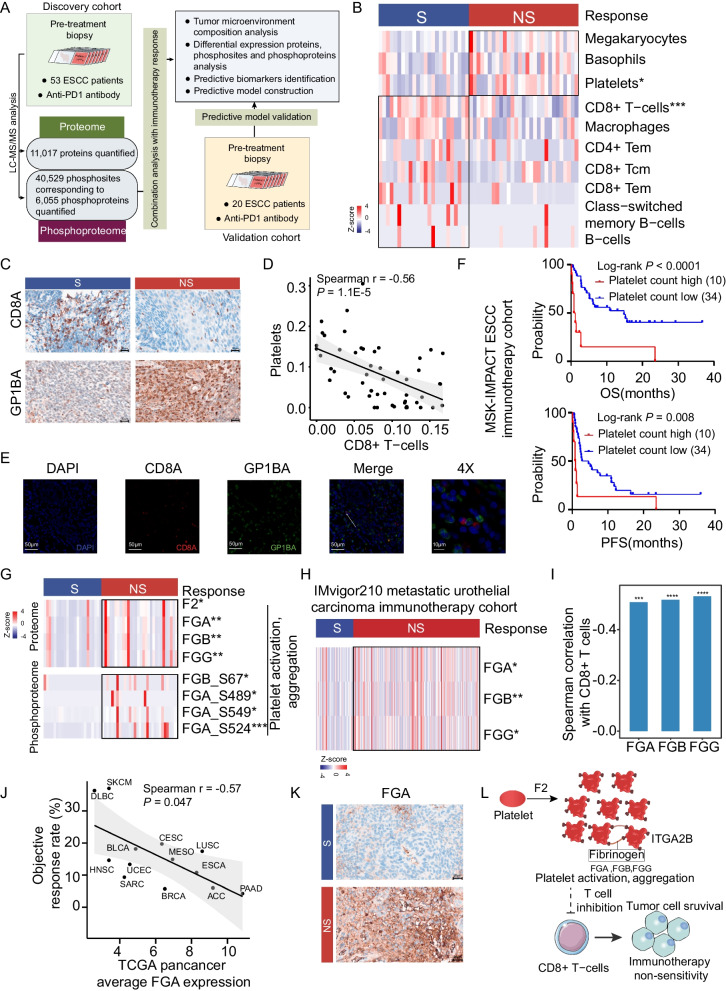
The association of platelets and ESCC immunotherapy response. **A** Overview of the workflow of proteomic profiling of ESCC immunotherapy cohort. **B** Heatmap of different abundance of xCell score between S and NS groups. **C** The representative images of immunohistochemistry (IHC) staining of CD8A and GP1BA expression in S and NS groups. The scale bar indicates 20 μm. **D** Spearman correlation analysis between CD8+ T-cells xCell score and platelets xCell score. *P* value was from two-sided Spearman correlation test. **E** Detection of CD8A and GP1BA in ESCC tumor tissue by multi-color IHC staining. Representative data from ESCC patients were shown. The scale bar indicates 50 or 10 μm. **F** Kaplan–Meier plots showing significant association of blood platelets counts with overall survival (OS) (upper) and progression-free survival (PFS) (bottom) in the MSK-IMPACT ESCC immunotherapy cohort. **G** The heatmap displaying the differential expression of proteins and their phosphosites involved in platelet activation, aggregation pathway between the S and NS groups. **H** The heatmap showing the differential expression of proteins involved in platelet activation, aggregation pathway in the IMvigor210 metastatic urothelial carcinoma immunotherapy cohort between the S and NS groups. **I** The Spearman correlation between the proteins involved in platelet activation, aggregation pathway and CD8+ T cells xCell score. *P* value was from two-sided Spearman correlation test. **J** Correlation between FGA protein expression and immunotherapy objective response rate in the TCGA pan-cancer cohort. *P* value was calculated by two-sided Spearman correlation test. **K** The qualification of FGA stained by IHC in the representative samples in the S and NS groups. The scale bar indicates 20 μm. **L** Systematic diagram summarizing the impact of the mechanism underlying ESCC patients with platelet activation is associated with immunotherapy non-sensitivity

## Findings

xCell analysis [7] found significantly higher platelets and CD8+ T cells level in NS and S group, respectively (Fig. 1B, C, Additional file 1: Fig. S1A, B and Table S2). Among all cell types, platelets exhibited the highest negative correlation with CD8 + T cells, and there was a potential direct physical interaction between platelets and CD8+ T cells (Fig. 1D, E and Additional file 1: Fig. S1C). NS group had significantly higher blood platelet count than S group (Additional file 1: Fig. S1D). Higher blood platelet count was related to shorter overall survival (OS) and progression free survival (PFS) in MSK-IMPACT ESCC immunotherapy cohort (log rank test *P* < 0.05) (Fig. 1F) [8]. Based on these findings, we speculated platelets might cause ESCC immunotherapy resistance by impairing CD8+ T cells function.

To further explore the connection of platelets and CD8+ T cells, we performed comparative analysis and found 298 significantly differential expression proteins (DEPs) between S and NS groups (Additional file 1: Fig. S1E). Pathway enrichment indicated that platelet activation and formation of fibrin clot pathway were enriched in NS group, and the upregulation of antigen processing and presentation, T cell receptor signaling pathway and fatty acid metabolism in S group, as well as molecules involved in these pathways at protein and phosphoprotein level (Additional file 1: Fig. S1F, G). GSEA analysis also showed platelet activation, aggregation pathway was significantly enriched in non-sensitive group (Additional file 1: Fig. S1H). The proteins involved in platelet activation such as F2, FGA, FGB and FGG were significantly upregulated in non-sensitive group, as well as phosphoprotein level (Fig. 1G and Additional file 1: Fig. S1I). Additionally, we also observed the similar expression of FGA, FGB and FGG in IMvigor210 metastatic urothelial carcinoma immunotherapy cohort (Fig. 1H) [9]. All of them showed significantly negative correlation with CD8+ T cells level (Fig. 1I). Among them, FGA expression exhibited significantly negative association with immunotherapy ORR across tumor types based on the TCGA pan-cancer mRNA expression datasets (Fig. 1J, K and Additional file 1: Fig. S1J) [10]. Overall, these results indicated that platelet activation attenuated immunotherapy response via inhibiting the immune effect of CD8+ T cells through a potential physical interaction (Fig. 1L).

**Fig. 2:**
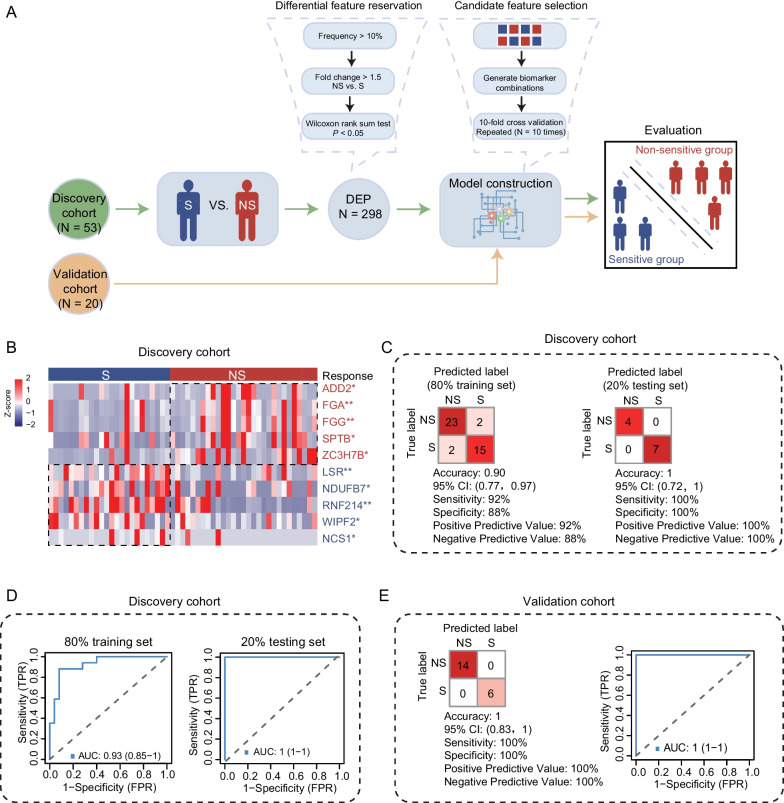
The construction and validation of predictive model for immunotherapy response. **A** Diagram describing a construction and validation of the predictive model for sensitive (S) and non-sensitive (NS) groups. **B** The heatmap displaying the 10 signatures that discriminate S and NS for ESCC immunotherapy in the discovery cohort. **C** Classification error matrix using logistic regression classifier of 80% training set and 20% testing set in the discovery cohort based on the 10 signatures combination. The number of samples identified is noted in each box. **D** ROC curves showing the predictive effect of this model in the 80% training set and 20% testing set of the discovery cohort. **E** Classification error matrix and ROC curve showing high sensitivity and specificity of the 10 signatures in the independent ESCC immunotherapy validation cohort

We next set out to determine whether the DEPs between S and NS groups could distinguish sensitive patients from non-sensitive patients in response to immunotherapy (Fig. 2A). We randomized discovery cohort into a training set (80%, N = 42) and a testing set (20%, N = 11). Based on the DEPs, we finally screened 10 signatures (including ADD2, FGA, FGG, SPTB, ZC3H7B, LSR, NDUFB7, RNF214, WIPF2 and NCS1) with high accuracy (0.90), sensitivity (92%) and specificity (88%) on training set, and 1, 100% and 100% on testing set (Additional file 1: Supplementary methods). The receiver operating characteristic (ROC) curves showed high predictive power of the model with area under curve (AUC) of 0.93 and 1 on training and testing sets, respectively. Furthermore, the model was also validated in an independent validation cohort (N = 20), including 6 S patients and 14 NS patients. Notably, the model also achieved high accuracy (1), sensitivity (100%) and specificity (100%) with AUC of 1 (Fig. 2B–E and Additional file 1: Fig. S2A–G).

Overall, the comprehensive proteomic analysis described an atlas of immunotherapy in ESCC. The activation of platelets in ESCC tumor microenvironment could decrease the anti-tumor efficacy of CD8+ T cells through a potential direct physical interaction, causing resistance to immunotherapy. Finally, we screened 10 biomarkers and constructed predictive model for predicting ESCC immunotherapy response, which could distinguish S patients from NS patients and contributed to personalized immunotherapy of ESCC patients.

### Supplementary Information

**Additional file 1.** Supplemental file for detailed methods and results.

## Data Availability

The data used and/or analysed during the current study are available from corresponding author on reasonable request. The accession number for the MS proteomics data reported in this paper is iProX repository (www.iprox.cn) [11]: the project ID IPX0006917000 (https://www.iprox.cn/page/PSV023.html;?url=1695357841577UV8p, password: 0lPT).

## Abbreviations

ESCC: Esophageal squamous cell carcinoma
MS: Mass spectrometry
ITP: Immune thrombocytopenia
S: Sensitive
NS: Non-sensitive
ORR: Objective response rate
OS: Overall survival
PFS: Progression free survival
DEPs: Differential expression proteins
ROC: The receiver operating characteristic
AUC: Area under the curve
