# Supplementary material for: Proteomic characterization of esophageal squamous cell carcinoma response to immunotherapy reveals potential therapeutic strategy and predictive biomarkers
Source: J Hematol Oncol. 2024 Mar 15;17:11. doi: 10.1186/s13045-024-01534-9 (PMC10943778; doi:10.1186/s13045-024-01534-9)
Supplement: Supplementary file 1 — Additional file 1. Supplemental file for detailed methods and results. [file 13045_2024_1534_MOESM1_ESM.docx]

Content

[Supplementary methods 2](#_Toc15688)

[Samples collection 2](#_Toc8261)

[Sample preparation 3](#_Toc7631)

[Protein extraction and trypsin digestion 3](#_Toc15393)

[The enrichment of phosphorylated peptides 4](#_Toc15627)

[Proteome and phosphoproteome analysis by LC-MS/MS analysis 4](#_Toc1184)

[Peptide and protein identification 6](#_Toc29972)

[Label-free-based MS quantification of proteins 6](#_Toc7051)

[Immune cell type composition 7](#_Toc6239)

[Differential protein and pathway analysis between S and NS group 7](#_Toc2600)

[Construction and validation of predictive models for ESCC immunotherapy response 7](#_Toc4401)

[Immunohistochemistry staining and evaluation 8](#_Toc26456)

[Immunofluorescence microscopy 8](#_Toc25041)

[Supplementary figures 10](#_Toc32645)

[Figure S1. The differential analysis between S and NS group in ESCC immunotherapy cohort 10](#_Toc13117)

[Figure S2. The introduction of additional DEPs of predictive model for ESCC immunotherapy response 12](#_Toc5391)

[Supplementary tables 13](#_Toc20059)

[Table S1: The baseline characteristics of ESCC immunotherapy discovery cohort 13](#_Toc17695)

[Tables S2: The sample composition data of different cell types. 14](#_Toc31757)

[References 18](#_Toc15364)

Supplementary methods

Samples collection

Treatment-naive archival formalin-fixed, paraffin-embedded (FFPE) tissues from ESCC patients were obtained and collected from 2019 to 2021; All samples were reviewed in the Department of Pathology, Shanghai Chest Hospital, Shanghai Jiao Tong University (Shanghai, R. P. China). Study was compliant with the ethical standards of Helsinki Declaration and was approved by the institutional review board. Written informed consent was obtained from each patient before any study-specific investigation was conducted.

This study consisted of 73 ESCC patients treated with first-line camrelizumab-based immunotherapy regimen. All the chemotherapy regimens were given at standard dosing as described in previous studies. Patients received two cycles of drug treatment, in each 21-day cycle, the following were administered intravenously: camrelizumab (200 mg) on day 1, nab-paclitaxel (260 mg/m2) on day 1, and carboplatin (area under the curve 5; 5 mg/mL/min) on day 1 [1]. After first two treatment cycles, all samples were histologically scored by three expert digestive system pathologists (J.W., C.X., and Y.H) according to the tumor regression grade (TRG) assessment criteria through measurement of the percentage of residual viable tumor on the resected tumor specimen after immunotherapy using previously reported methods [1, 2], and respectively grouped into: TRG-I, without residual tumor; TRG-II, 1% - 10% residual tumor; TRG-III, 11% - 50% residual tumor; TRG-IV, > 50% residual tumor. Patients with TRG-I who were the pathological complete response (pCR) due to the absence of residual viable tumor were defined as sensitive (S), and those with TRG-II-TRG-IV were defined as non-sensitive (NS), consistent with the previous definition [1]. The discovery cohort included 24 sensitive patients (TRG-I, N = 24) and 29 non-sensitive patients (TRG-II, N = 9; TRG-III, N = 8; TRG-IV, N = 12). The validation cohort comprised 6 sensitive patients (TRG-I, N=6) and 14 non-sensitive patients (TRG-II, N = 8; TRG-III, N = 3; TRG-IV, N = 3).

FFPE tissues with at least 80% tumor purity were collected from a total of 73 therapy-naïve patients with ESCC before initial camrelizumab-based immunotherapy. The PD-L1 expression of all tumor samples was assessed by a PD-L1 immunohistochemistry kit (6E8 antibody: Abcam) and characterized according to tumor proportion score (TPS), consistent with the clinical trial [3]. A tumor proportion score of 1% was used as the cutoff of PD-L1 positive and negative. The IHC staining intensity of PD-L1 less than 1% was defined as PD-L1 negative (PD-L1-), while PD-L1-postive (PD-L1+) was defined with at least 1% PD-L1-positive tumor cells according to the reported method [4]. For the baseline blood platelet count of ESCC patients included in our cohort, a platelet count was with a range of 115-425 × 10^9^/L, which was higher than the diagnosis criteria (100 × 10^9^/L) of ITP [5, 6]. Therefore, according to the clinical characteristics of ESCC patients in our study, we confirmed that there was no ESCC patients diagnosed with ITP in our cohort. The detailed clinical characteristics were shown in Supplementary table 1.

Sample preparation

The biopsy tumor FFPE samples derived from 73 therapy-naïve ESCC patients were collected, and the tumor regions were determined by pathological examination. For proteomic and phosphoproteomic sample preparation, sections (10 μm thick) from FFPE blocks were macro-dissected, deparaffinized with xylene, and washed with ethanol. The ethanol was removed completely and the sections were left to air-dry. For this purpose, a hematoxylin-stained section of the same tumor was used as reference. Areas containing 80% or more tumor were examined independently by three expert gastrointestinal pathologists (J.W., C.X., and Y.H). Each sample was assigned a new research ID, and the patient’s name or medical record number used during hospitalization was de-identified.

Protein extraction and trypsin digestion

The biopsy tumor FFPE samples for each sample was lysed in TCEP buffer (2% deoxycholic acid sodium salt, 40 mM 2-Chloroacetamide, 100 mM Tris-HCl, 10 mM Tris(2-chloroethyl) phosphate, 1 mM PFSM, 1 mM Cocktail, pH 8.5) supplemented with protease inhibitors and phosphatase at 99 °C for 30 min. After cooling to room temperature, trypsin (Promega, Madison, WI, USA, #V5280) was added and digested for 18 h at 37˚C. 10% formic acid was added and vortex for 3 min, followed by sedimentation for 5 min (12,000 g). Next, a new 1.5 mL tube with extraction buffer (0.1% formic acid in 50% acetonitrile) was used to extract the supernatant (vortex for 3 min, followed by 12,000 g of sedimentation for 5 min). Collected supernatant was divided into two part and dried using a speed-vac, one for proteome and other one for phosphoproteome.

The enrichment of phosphorylated peptides

Tryptic peptides were used for phosphopeptide enrichment using a High-Select Fe-NTA kit (Thermo Fisher Scientific, Rockford, IL, USA, #A32992) according to the kit manual and a previous report [7] with some modifications. In brief, peptides were suspended in binding/wash buffer (contained in the enrichment kit) and mixed with the equilibrated resins. The peptide-resin mixture was incubated for 30 min with three gentle blows at room temperature. Following incubation, the resins were washed thrice with binding/wash buffer and twice with water. The enriched peptides were eluted with elution buffer (contained in the enrichment kit) and immediately dried using a speed-vac at 30 °C for mass spectrometry analysis.

Proteome and phosphoproteome analysis by LC-MS/MS analysis

For the proteomic profiling of samples, peptides were analyzed on a Q-Exactive HFX Hybrid Quadrupole-Orbitrap Mass Spectrometer (Thermo Fisher Scientific, Rockford, IL, USA) coupled with a high-performance liquid chromatography system (EASY nLC 1200, Thermo Fisher). Dried peptide samples re-dissolved in Solvent A (0.1% FA in water) were loaded to a 2-cm self-packed trap column (100-μm inner diameter, 3 m ReproSil-Pur C18-AQ beads, Dr. Maisch GmbH) using Solvent A and separated on a 150-μm-inner-diameter column with a length of 15 cm (1.9 μm ReproSil-Pur C18-AQ beads, Dr. Maisch GmbH) over a 150 min gradient (Solvent A: 0.1% FA in water; Solvent B: 0.1% FA in 80% ACN) at a constant flow rate of 600 nL/min (0–150 min, 0 min, 4% B; 0–10 min, 4–15% B; 10–125 min, 15–30% B; 125–140 min, 30–50% B; 140–141 min, 50–100% B; 141–150 min, 100% B). The eluted peptides were ionized under 2.0 kV and introduced into mass spectrometer). MS was performed under a data-dependent acquisition mode. For the MS1 Spectra full scan, ions with m/z ranging from 300 to 1,400 were acquired by Orbitrap mass analyzer at a high resolution of 120,000. The automatic gain control (AGC) target value was set as 3E6. The maximal ion injection time was 80 ms. MS2 Spectra acquisition was performed in the ion trap mode at a rapid speed. Precursor ions were selected and fragmented with higher energy collision dissociation (HCD) with a normalized collision energy of 27%. Fragment ions were analyzed by the ion trap mass analyzer with the AGC target at 5E4. The maximal ion injection time of MS2 was 20 ms. Peptides that triggered MS/MS scans were dynamically excluded from further MS/MS scans for 25 s.

For the phosphoproteomic samples, peptides were analyzed on a Q Exactive HF-X Hybrid Quadrupole-Orbitrap Mass Spectrometer (Thermo Fisher Scientific) coupled with a high-performance liquid chromatography system (EASY nLC 1200, Thermo Fisher Scientific). Dried peptide samples re-dissolved in Solvent A (0.1% formic acid in water) were loaded onto a 2-cm self-packed trap column (100 μm inner diameter, 3 μm ReproSil-Pur C18-AQ beads, Dr. Maisch GmbH) using Solvent A and separated on a 150-μm-inner-diameter column with a length of 30 cm (1.9 μm ReproSil-Pur C18-AQ beads, Dr. Maisch GmbH) over a 150-min gradient (buffer A: 0.1% formic acid in water; buffer B: 0.1% formic acid in 80% ACN) at a constant flow rate of 600 nL/min (0-150 min, 0 min, 4% B; 0-9 min, 4-15% B; 9-129 min, 15-30% B; 129-140 min, 30-50% B; 140-141 min, 50-100% B; 141-150 min, 100% B). The eluted phosphopeptides were ionized and detected by a Q-Exactive HF-X Hybrid Quadrupole-Orbitrap mass spectrometry. Mass spectra were acquired over the scan range of m/z 300-1400 at a resolution of 120,000 (AUG target value of 3E+06 and maximum injection time 80 ms). For the MS2 scan, higher energy collision dissociation fragmentation was performed at a normalized collision energy of 30%. The MS2 AGC target was set to 5E+04 with a maximum injection time of 100 ms. The peptide mode was selected for monoisotopic precursor scan, and charge state screening was enabled to reject unassigned 1+, 7+, 8+, and > 8+ ions with a dynamic exclusion time of 40 s to discriminate against previously analyzed ions between ± 10 ppm. All data were acquired using Xcalibur software v2.2 (Thermo Fisher Scientific).

Peptide and protein identification

MS raw files were processed using the Firmiana proteomics workstation [8] (a one-stop proteomic cloud platform: http://www.firmiana.org). Briefly, raw files were searched against the NCBI human Refseq protein database using the Mascot search engine (version 2.3, Matrix Science Inc). The mass tolerances were: 20 ppm for precursor and 50 mmu for product ions collected by Q Exactive HF-X. Up to two missed cleavages were allowed. The database searching of proteome considered cysteine carbamidomethylation as a fixed modification, and N-acetylation, and oxidation of methionine as variable modifications. The database searching of phosphoproteome considered cysteine carbamidomethylation as a fixed modification, and N-acetylation, phosphorylation of serine, threonine and tyrosine, and oxidation of methionine as variable modifications. Precursor ion score charges were limited to +2, +3, and +4. For the quality control of protein identification, the target-decoy-based strategy was applied to confirm the FDR of both peptide and protein, which was lower than 1%. Percolator was used to obtain the quality value (q-value), validating the FDR (measured by the decoy hits) of every peptide-spectrum match (PSM), which was lower than 1%. Subsequently, all the peptides shorter than seven amino acids were removed. The cutoff ion score for peptide identification was 20. All the PSMs in all fractions were combined to comply with a stringent protein quality control strategy. We employed the parsimony principle and dynamically increased the q-values of both target and decoy peptide sequences until the corresponding protein FDR was less than 1%. Finally, to reduce the false positive rate, the proteins with at least one unique peptide were selected for further investigation. Phosphosites were confirmed by the PhosphoSitePlus database (<https://www.phosphosite.org>).

Label-free-based MS quantification of proteins

The one-stop proteomic cloud platform “Firmiana” was further employed for protein quantification. Identification results and the raw data from the mzXML file were loaded. Then for each identified peptide, the extracted-ion chromatogram (XIC) was extracted by searching against the MS1 based on its identification information, and the abundance was estimated by calculating the area under the extracted XIC curve. For protein abundance calculation, the nonredundant peptide list was used to assemble proteins following the parsimony principle. The protein abundance was estimated using a traditional label-free, intensity-based absolute quantification (iBAQ) algorithm [9], which divided the protein abundance (derived from identified peptides’ intensities) by the number of theoretically observable peptides. We built a dynamic regression function based on the commonly identified peptides in tumor samples. According to correlation value R^2^, Firmiana chose linear or quadratic functions for regression to calculate the retention time (RT) of corresponding hidden peptides, and to check the existence of the XIC based on the m/z and calculated RT. Subsequently, the fraction of total (FOT), a relative quantification value was defined as a protein’s iBAQ divided by the total iBAQ of all identified proteins in one experiment, and was calculated as the normalized abundance of a particular protein among experiments. Finally, the FOT was further multiplied by 10^5^ for ease of presentation, and FOTs less than 10^-5^ were replaced with 10^-5^ to adjust extremely small values [10].

Immune cell type composition

The abundance of 64 different cell types were computed via xCell based on proteomic profiles [11]. The Supplementary table 2 contains the final score computed by xCell of different cell types.

Differential protein and pathway analysis between S and NS group

The protein expression matrices were used to perform the differential expression analysis of the sensitive group (S) and the non-sensitive group (NS). The overrepresented proteins of S and NS were defined as proteins differentially expressed in the S and NS groups (NS/S > 1.5 or < 0.67); the significantly differentially expressed proteins was defined as proteins with more than 1.5-fold change and Wilcoxon rank-sum test with a p value cutoff (*P* < 0.05). Pathway enrichment analysis of the overrepresented proteins was performed by CPDB databases. The same method was also applied to phosphoproteins and phosphorylation sites between S and NS groups for the differential analysis.

Construction and validation of predictive models for ESCC immunotherapy response

Multiple logistic regression analysis was used to construct the therapeutic response prediction model based on the significantly differentially expressed proteins between S and NS groups in R software v3.5.1. The 53 ESCC patients in discovery cohort were randomly divided into 80% of individuals (the training set) and the remaining 20% (the testing set). And backward stepwise method was utilized to feature selection in the training set, resulting in 9 signatures. Based on the 9 signatures, we applied 10-fold cross-validation to training set yield predictive model to predict ESCC immunotherapy response by using the Caret package (https://cran.r-project.org/web/packages/caret/index.html). Moreover, the diagnostic ability of these model was validated in an independent validation cohort including 20 ESCC patients. To enhance the predictive power of the predictive model, we also introduced additional DEPs and assessed the performance of predictive model on discovery cohort (training and testing sets) and validation cohort by the same method. We selected the proteins based on the criteria: the introduced DEPs combination could achieve higher accuracy in training set, testing set and validation cohort in sequence compared with the previous combination, respectively. Finally, we introduced 1 DEP (NCS1) to form the 10 DEPs combination to predict ESCC immunotherapy response.

Immunohistochemistry staining and evaluation

A standard immunohistochemistry (IHC) protocol was followed to stain the tumor tissue samples using the rabbit polyclonal antibody against CD8A (Signalway Antibody, catalog No: 48750-1), the rabbit polyclonal antibody against GP1BA (Signalway Antibody, catalog No: 36512) and the rabbit polyclonal antibody against FGA (Signalway Antibody, catalog No: 53871-1). IHC evaluation was analyzed using an IHC profiler compatible plugin with integrated options for the quantitative analysis of digital IHC images stained for cytoplasmic or nuclear proteins [12]. Moreover, the intensity of the cytoplasmic staining and the percentage of positively stained tumor cells were also scored numerically.

Immunofluorescence microscopy

Formalin-fixed paraffin-embedded tissue sections (4 μm) from ESCC treatment-naive patients were collected and performed immunofluorescence staining according to the previously described method [13]. Briefly, Tumor FFPE slides were baked at 50 °C overnight, deparaffinized in xylene and rehydrated in decreasing concentration of ethanol (100%, 90%, 70%, 50% and dH_2_O). Sample slides were incubated in pH 6 or pH 9 buffers at 95 °C for 30 min for antigen retrieval, then in 3% hydrogen peroxide for 15 min for permeabilization and in serum-free protein block solution for 30 min for blocking non-specific binding. Then, tumor biopsies were stained with primary anti-GP1BA antibody (Signalway Antibody, catalog No: 36512) and anti-CD8A antibody (Signalway Antibody, catalog No: 36512), followed incubated by goat anti-rabbit IgG (H+L) Highly Cross-Adsorbed Secondary Antibody, Alexa Fluor 488 (Abcam, catalog No: ab150077) or Cyanine3 (Invitrogen, catalog No: A10520). Nucleic acid was stained with DAPI (Invitrogen, catalog No: P36931). Finally, stained slides were mounted using Prolong Gold Antifade and slides were imaged with a confocal microscope.

Supplementary figures


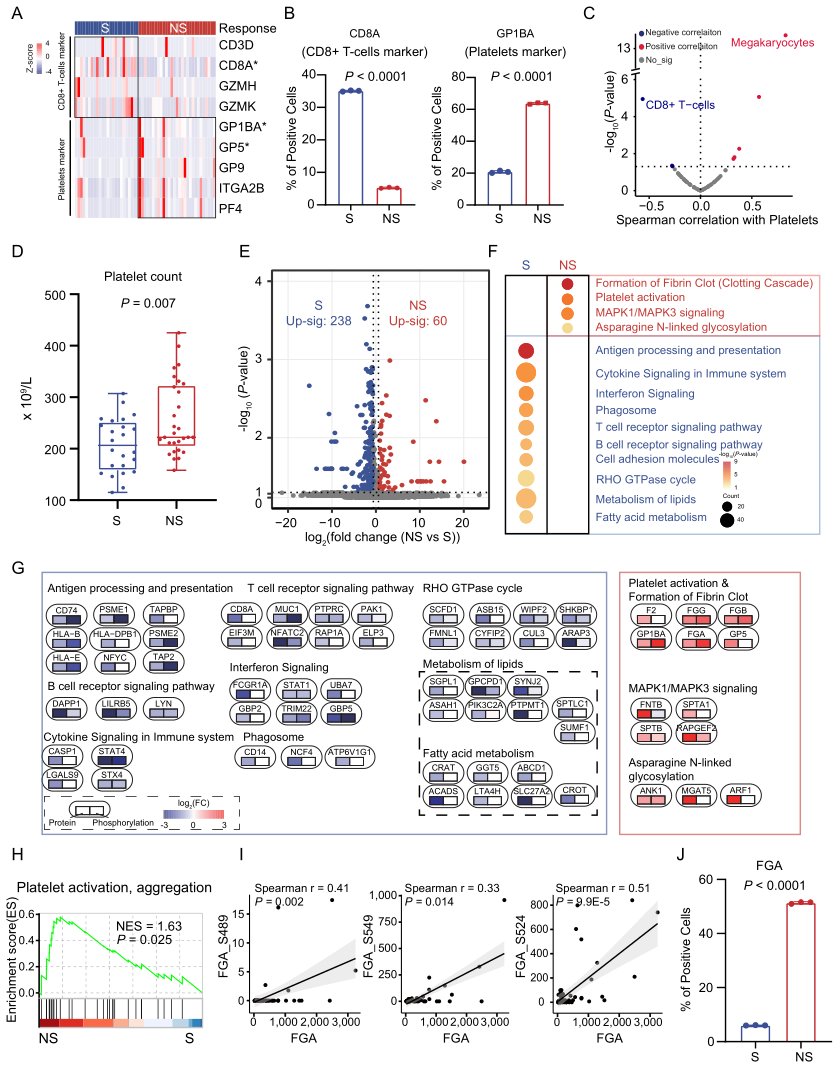


Figure S1. The differential analysis between S and NS group in ESCC immunotherapy cohort**. A** Heatmap showing the abundance of CD8+T cells markers and platelets markers in S and NS groups. **B** The boxplots displaying the qualification of markers (CD8A and GP1BA) of CD8+ T-cells and platelets stained by immunohistochemistry (IHC) in the representative samples in the S and NS groups (n = 3 independent experiments, two-sided Student’s t test, mean ± SD). **C** Volcano plot showing the correlation of CD8+ T-cell xCells scores with other xCell signature scores. *P*-value was from two-sided Spearman correlation test. **D** The blood platelet count of ESCC patients before receiving anti-PD1 immunotherapy and its difference between S and NS groups. *P*-value was calculated by two-sided Student’s t test. **E** Volcano plot showing the differential expression of proteins in S and NS groups (two-sided Wilcoxon rank-sum test). **F** Bubble plot showing the pathway enrichment of S and NS groups. *P*-value was calculated according to the hypergeometric test. **G** Diagram illustrating the differentially expressed proteins (left) and phosphoproteins (right) and signaling cascades involved in S and NS groups. The little heatmap under each protein depicted the fold change of S and NS groups. **H** GSEA enrichment plot of platelet activation, aggregation in the NS group. *P*-value from Phenotype-based permutation test. **I** Spearman correlation analysis between FGA protein expression and its phosphosites (two-sided Spearman correlation test). **J** The boxplot showing the qualification of FGA stained by immunohistochemistry (IHC) in the representative samples in the S and NS groups (n = 3 independent experiments, two-sided Student’s t test, mean ± SD).


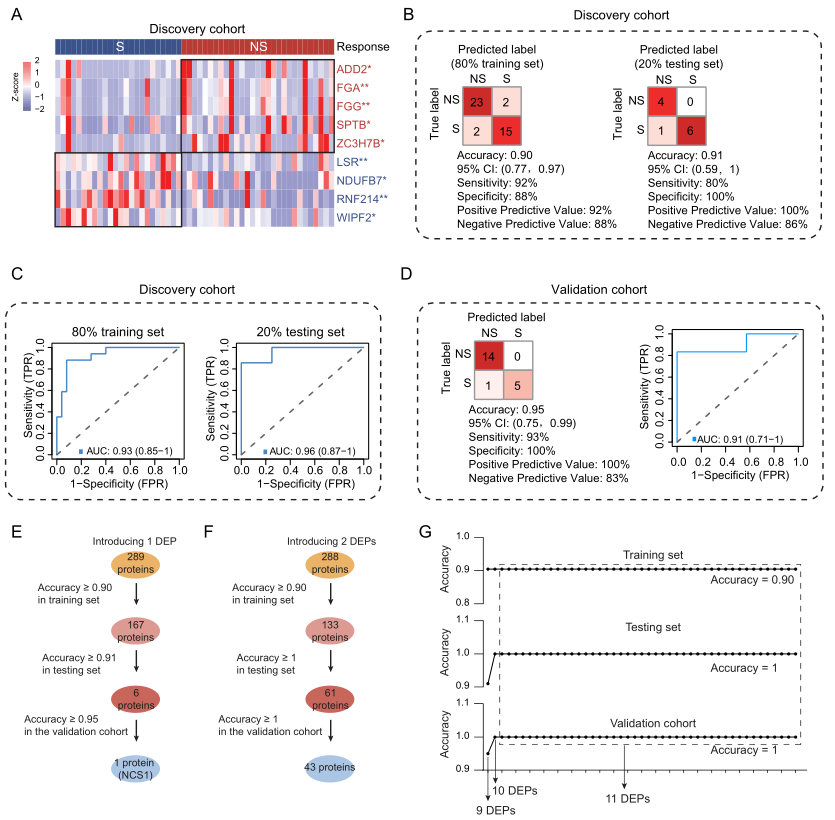


Figure S2. The introduction of additional DEPs of predictive model for ESCC immunotherapy response**. A** The heatmap displaying the 9 signatures selected by stepwise logistic regression method, which discriminate S and NS for ESCC immunotherapy in the discovery cohort. **B** Classification error matrix using logistic regression classifier of training set and testing set based on the 9 signature proteins in the discovery cohort. The number of samples identified is noted in each box. **C** ROC curves showing the predictive effect of this model in the training set and testing set of the discovery cohort based on the 9 signature proteins. **D** Classification error matrix and ROC curve showing high sensitivity and specificity of the 9 signatures in the independent ESCC immunotherapy validation cohort. **E-F** The criteria of introduction 1 DEP or 2DEPs to the 9 DEPs combination. **G** The accuracy of predictive model in training set, testing set and validation cohort in 9 DEPs combination, 10 DEPs combination and 11 DEPs combination.

Supplementary tables

Table S1: The baseline characteristics of ESCC immunotherapy discovery cohort.

| Study ID | Age  (years) | Gender | Smoking | Drinking | Tumor  location | Histological grade | PD-L1  expression | cStage | TRG | Response |
| --- | --- | --- | --- | --- | --- | --- | --- | --- | --- | --- |
| 1 | >60 | Male | Yes | Yes | Middle | Poorly differentiated | Positive | III | I | S |
| 2 | >60 | Male | Yes | Yes | Middle | Moderately differentiated | Positive | III | IV | NS |
| 3 | >60 | Male | Yes | Yes | Upper | Moderately differentiated | Negative | III | II | NS |
| 4 | >60 | Male | Yes | Yes | Middle | Moderately differentiated | Negative | III | I | S |
| 5 | >60 | Male | Yes | Yes | Upper | Poorly differentiated | Negative | III | I | S |
| 6 | >60 | Male | Yes | Yes | Middle | Poorly differentiated | Negative | III | I | S |
| 7 | ≤60 | Male | Yes | Yes | Upper | Poorly differentiated | Negative | III | I | S |
| 8 | >60 | Male | Yes | No | Lower | Moderately differentiated | Positive | III | I | S |
| 9 | >60 | Male | Yes | No | Upper | Moderately differentiated | Positive | Iva | II | NS |
| 10 | >60 | Male | Yes | Yes | Middle | Poorly differentiated | Negative | IVa | IV | NS |
| 11 | >60 | Female | No | No | Middle | Well differentiated | Positive | IVa | I | S |
| 12 | >60 | Female | No | No | Middle | Poorly differentiated | Positive | III | I | S |
| 13 | >60 | Male | Yes | No | Middle | Poorly differentiated | Negative | IVa | III | NS |
| 14 | >60 | Male | Yes | Yes | Middle | Moderately differentiated | Negative | III | I | S |
| 15 | >60 | Male | Yes | No | Middle | Poorly differentiated | Negative | III | I | S |
| 16 | ≤60 | Male | Yes | Yes | Middle | Moderately differentiated | Negative | IVa | IV | NS |
| 17 | >60 | Male | Yes | Yes | Upper | Poorly differentiated | Positive | III | I | S |
| 18 | ≤60 | Male | Yes | Yes | Upper | Moderately differentiated | Positive | IVa | III | NS |
| 19 | >60 | Male | Yes | No | Middle | Moderately differentiated | Negative | III | I | S |
| 20 | >60 | Female | No | No | Middle | Poorly differentiated | Positive | III | I | S |
| 21 | >60 | Male | Yes | Yes | Middle | Moderately differentiated | Negative | III | III | NS |
| 22 | >60 | Male | Yes | No | Middle | Poorly differentiated | Positive | III | I | S |
| 23 | >60 | Male | Yes | Yes | Middle | Poorly differentiated | Positive | III | II | NS |
| 24 | >60 | Male | Yes | No | Middle | Poorly differentiated | Negative | III | I | S |
| 25 | >60 | Male | No | No | Middle | Moderately differentiated | Negative | IVa | I | S |
| 26 | ≤60 | Male | Yes | Yes | Upper | Moderately differentiated | Positive | III | IV | NS |
| 27 | ≤60 | Male | Yes | Yes | Upper | Moderately differentiated | Negative | III | IV | NS |
| 28 | >60 | Male | Yes | Yes | Middle | Moderately differentiated | Negative | III | I | S |
| 29 | >60 | Male | Yes | Yes | Upper | Poorly differentiated | Positive | III | I | S |
| 30 | ≤60 | Female | No | No | Upper | Poorly differentiated | Negative | III | I | S |
| 31 | >60 | Male | Yes | No | Middle | Moderately differentiated | Positive | IVa | III | NS |
| 32 | >60 | Female | No | No | Middle | Moderately differentiated | Negative | III | II | NS |
| 33 | >60 | Male | Yes | Yes | Middle | Moderately differentiated | Negative | III | II | NS |
| 34 | >60 | Male | Yes | No | Middle | Poorly differentiated | Positive | IVa | I | S |
| 35 | >60 | Male | Yes | No | Middle | Moderately differentiated | Positive | III | I | S |
| 36 | >60 | Male | Yes | Yes | Lower | Poorly differentiated | Positive | III | I | S |
| 37 | >60 | Male | Yes | Yes | Upper | Poorly differentiated | Positive | III | II | NS |
| 38 | ≤60 | Male | Yes | No | Middle | Moderately differentiated | Positive | IVa | II | NS |
| 39 | ≤60 | Male | Yes | Yes | Upper | Moderately differentiated | Positive | III | IV | NS |
| 40 | >60 | Male | Yes | Yes | Upper | Poorly differentiated | Positive | IVa | III | NS |
| 41 | >60 | Male | Yes | Yes | Lower | Poorly differentiated | Positive | IVa | IV | NS |
| 42 | ≤60 | Male | Yes | Yes | Middle | Moderately differentiated | Positive | IVa | IV | NS |
| 43 | >60 | Female | No | No | Middle | Moderately differentiated | Negative | IVa | I | S |
| 44 | ≤60 | Male | Yes | Yes | Middle | Poorly differentiated | Negative | III | III | NS |
| 45 | >60 | Male | Yes | Yes | Upper | Moderately differentiated | Positive | III | II | NS |
| 46 | >60 | Female | No | No | Middle | Poorly differentiated | Negative | III | II | NS |
| 47 | >60 | Male | Yes | Yes | Lower | Moderately differentiated | Positive | III | IV | NS |
| 48 | >60 | Male | Yes | Yes | Middle | Poorly differentiated | Positive | IVa | IV | NS |
| 49 | >60 | Female | No | No | Middle | Moderately differentiated | Positive | IVa | IV | NS |
| 50 | >60 | Male | Yes | Yes | Middle | Well differentiated | Positive | IVa | I | S |
| 51 | >60 | Male | Yes | Yes | Middle | Poorly differentiated | Positive | III | III | NS |
| 52 | >60 | Male | Yes | No | Upper | Poorly differentiated | Positive | III | III | NS |
| 53 | >60 | Male | Yes | Yes | Lower | Moderately differentiated | Positive | IVa | IV | NS |

## Tables S2: The sample composition data of different cell types.

| Cell type | 1 | 2 | 3 | 4 | 5 | 6 | 7 | 8 | 9 | 10 | 11 | 12 | 13 | 14 |
| --- | --- | --- | --- | --- | --- | --- | --- | --- | --- | --- | --- | --- | --- | --- |
| NK cells | 0 | 0 | 0 | 0.0031 | 0 | 0 | 0 | 0 | 0 | 0 | 0 | 0 | 0 | 0 |
| Megakaryocytes | 0.068 | 0.0816 | 0.4905 | 0.1097 | 0.1506 | 0.0374 | 0.029 | 0 | 0.1184 | 0.0493 | 0.0508 | 0.0396 | 0.2551 | 0.0332 |
| Basophils | 0.2336 | 0.1397 | 0.3263 | 0.2734 | 0.1539 | 0.1447 | 0.1433 | 0 | 0.1562 | 0 | 0.1214 | 0.0829 | 0.2181 | 0 |
| Neutrophils | 0.0794 | 0.0456 | 0.0198 | 0.0003 | 0.0894 | 0.0406 | 0.0425 | 0 | 0.0057 | 0.0077 | 0.0036 | 0.0074 | 0.1097 | 0.0645 |
| Endothelial cells | 0.0516 | 0.1042 | 0 | 0.0983 | 0.1266 | 0.0926 | 0 | 0.0787 | 0.0878 | 0.0333 | 0 | 0.0549 | 0.0961 | 0.0969 |
| mv Endothelial cells | 0.0534 | 0.0928 | 0 | 0.0724 | 0 | 0.1014 | 0.06 | 0.0036 | 0.0278 | 0.0332 | 0.0248 | 0.0351 | 0.0207 | 0.0216 |
| CLP | 0.1002 | 0.0976 | 0.1036 | 0.0371 | 0 | 0.0802 | 0.0777 | 0.1038 | 0.1104 | 0.1483 | 0.1235 | 0.0974 | 0.0396 | 0.0571 |
| Hepatocytes | 0.0179 | 0.0267 | 0.0288 | 0.0487 | 0.0502 | 0 | 0.0007 | 0.0101 | 0.0406 | 0 | 0.0103 | 0.0181 | 0.0368 | 0.0171 |
| ly Endothelial cells | 0.0903 | 0.1432 | 0.0854 | 0.1413 | 0.126 | 0.1521 | 0.018 | 0.0753 | 0.0944 | 0.0419 | 0.0169 | 0.0441 | 0.15 | 0.0576 |
| Melanocytes | 0.0155 | 0 | 0.0314 | 0.0133 | 0.0002 | 0.0265 | 0.0225 | 0.0186 | 0.0065 | 0.0436 | 0.0175 | 0.0189 | 0.0178 | 0.0166 |
| naive B-cells | 0 | 0 | 0 | 0 | 0 | 0 | 0 | 0 | 0 | 0 | 0 | 0 | 0 | 0 |
| Myocytes | 0.0391 | 0.0443 | 0.0238 | 0 | 0.0467 | 0 | 0.0453 | 0.0008 | 0.0155 | 0.0426 | 0.0086 | 0.007 | 0.0382 | 0.005 |
| Th1 cells | 0.0747 | 0.0854 | 0.1801 | 0.1428 | 0.0043 | 0 | 0.1138 | 0.1187 | 0 | 0.1277 | 0.0767 | 0.0861 | 0.0678 | 0.0342 |
| Chondrocytes | 0.0445 | 0.2057 | 0 | 0.1952 | 0.2603 | 0.1758 | 0 | 0.0922 | 0.0537 | 0.1695 | 0.1233 | 0.0999 | 0.164 | 0.1631 |
| Neurons | 0 | 0.0102 | 0 | 0.0158 | 0.0008 | 0.0202 | 0.0206 | 0.0208 | 0.0184 | 0.0234 | 0.0134 | 0.0202 | 0.0114 | 0.0142 |
| NKT | 0.1542 | 0.0383 | 0.1718 | 0.0585 | 0.0726 | 0.3022 | 0.0481 | 0.1848 | 0 | 0.1685 | 0.3795 | 0.0593 | 0.1071 | 0.3976 |
| Monocytes | 0.1151 | 0.0377 | 0.0132 | 0 | 0.0282 | 0.0157 | 0.0717 | 0.0973 | 0.0638 | 0.0068 | 0.003 | 0.0628 | 0.0673 | 0.0153 |
| Epithelial cells | 0.019 | 0 | 0.0234 | 0 | 0 | 0.0657 | 0.081 | 0.1149 | 0.0875 | 0.0694 | 0.0984 | 0.0609 | 0.006 | 0.036 |
| HSC | 0.1534 | 0.2331 | 0.1937 | 0.0646 | 0.3734 | 0.2168 | 0.0083 | 0.1691 | 0.1052 | 0.2304 | 0.1894 | 0.2273 | 0.2934 | 0.3121 |
| StromaScore | 0.1066 | 0.0789 | 0 | 0.1589 | 0.1551 | 0.1405 | 0 | 0.131 | 0.1115 | 0.1491 | 0.011 | 0.1104 | 0.1621 | 0.1274 |
| aDC | 0.0678 | 0.1245 | 0.111 | 0.0992 | 0.1621 | 0.0671 | 0 | 0.1076 | 0.0815 | 0.1196 | 0.1039 | 0.0453 | 0.0203 | 0.0549 |
| Mesangial cells | 0.0072 | 0.0616 | 0 | 0.0541 | 0.0248 | 0.0085 | 0.0027 | 0.0225 | 0.0541 | 0.0079 | 0.0059 | 0 | 0.0273 | 0.033 |
| pDC | 0.0343 | 0 | 0 | 0.0311 | 0 | 0.0047 | 0.0236 | 0.0888 | 0.0588 | 0.0338 | 0.0328 | 0.0472 | 0 | 0.0023 |
| Skeletal muscle | 0.0285 | 0.076 | 0.0043 | 0.0308 | 0.0399 | 0.0278 | 0 | 0.011 | 0.0208 | 0.0267 | 0.0112 | 0.0348 | 0.022 | 0 |
| Keratinocytes | 0.0328 | 0 | 0.0835 | 0.0375 | 0.037 | 0.0447 | 0.1128 | 0.1202 | 0.1128 | 0.0762 | 0.081 | 0.0636 | 0.053 | 0.0371 |
| Mast cells | 0.0262 | 0.0287 | 0.0197 | 0.044 | 0.0364 | 0.0166 | 0.0167 | 0.017 | 0.0256 | 0.0128 | 0.0166 | 0.0154 | 0.0377 | 0.0041 |
| CD4+ naive T-cells | 0.0442 | 0.0625 | 0 | 0.1634 | 0.036 | 0.0008 | 0.0133 | 0 | 0 | 0 | 0 | 0.0191 | 0.0712 | 0.0762 |
| Sebocytes | 0.0108 | 0 | 0.018 | 0.0137 | 0.0075 | 0.0153 | 0.0194 | 0.0208 | 0.0181 | 0.017 | 0.0167 | 0.0131 | 0.0112 | 0.0144 |
| Fibroblasts | 0.098 | 0.0536 | 0 | 0.1923 | 0.1669 | 0.1082 | 0 | 0.1079 | 0.1116 | 0.1965 | 0.022 | 0.1381 | 0.155 | 0.123 |
| Adipocytes | 0.0635 | 0 | 0 | 0.0272 | 0.0168 | 0.0801 | 0 | 0.0755 | 0.0236 | 0.0683 | 0 | 0.0277 | 0.0731 | 0.0349 |
| Osteoblast | 0.0885 | 0.096 | 0.1696 | 0.1849 | 0.1589 | 0.2125 | 0.1502 | 0.0196 | 0.1478 | 0.2173 | 0.3141 | 0.2773 | 0.02 | 0.0769 |
| Pericytes | 0.0469 | 0.0019 | 0.0764 | 0.0375 | 0.0114 | 0.0778 | 0.0135 | 0.0725 | 0.0012 | 0 | 0 | 0.0469 | 0.0595 | 0 |
| Macrophages M2 | 0.0722 | 0.0051 | 0.0734 | 0.0227 | 0.0283 | 0 | 0.0188 | 0.035 | 0.0381 | 0.043 | 0.0872 | 0.0687 | 0.0306 | 0.1301 |
| Th2 cells | 0.1334 | 0.0244 | 0.0216 | 0.0065 | 0.1209 | 0 | 0 | 0 | 0.0664 | 0.0714 | 0.0733 | 0.0768 | 0.092 | 0.0886 |
| MicroenvironmentScore | 0.4837 | 0.5828 | 0.1704 | 0.3753 | 0.4035 | 0.4306 | 0.2718 | 0.3635 | 0.1749 | 0.2944 | 0.1279 | 0.3254 | 0.44 | 0.5345 |
| Erythrocytes | 0 | 0 | 0 | 0 | 0.0175 | 0 | 0 | 0 | 0 | 0 | 0 | 0.0053 | 0 | 0 |
| GMP | 0.0785 | 0.1512 | 0 | 0.1537 | 0.1098 | 0.1027 | 0.0589 | 0.079 | 0 | 0.1198 | 0.0664 | 0.1098 | 0.1082 | 0.0753 |
| CD4+ memory T-cells | 0.195 | 0.188 | 0.1027 | 0.2073 | 0.1168 | 0.1405 | 0.1451 | 0.1854 | 0.183 | 0.1286 | 0.0028 | 0.1577 | 0.1185 | 0.1729 |
| Astrocytes | 0 | 0.0136 | 0 | 0.0157 | 0 | 0 | 0 | 0 | 0.0675 | 0 | 0 | 0 | 0 | 0 |
| ImmuneScore | 0.3771 | 0.5039 | 0.1704 | 0.2164 | 0.2484 | 0.2902 | 0.2718 | 0.2324 | 0.0634 | 0.1454 | 0.1169 | 0.215 | 0.2779 | 0.4071 |
| CD8+ naive T-cells | 0.0931 | 0.1617 | 0.1426 | 0.242 | 0.1831 | 0.1835 | 0.1776 | 0.1199 | 0.0877 | 0.1973 | 0.1283 | 0.1929 | 0.1202 | 0.0032 |
| iDC | 0.0257 | 0.1629 | 0.0534 | 0.1741 | 0.0284 | 0.0847 | 0.0618 | 0.0384 | 0.0173 | 0.072 | 0.0293 | 0.0767 | 0.0528 | 0.206 |
| Eosinophils | 0.1133 | 0.3215 | 0.1383 | 0.1076 | 0.1451 | 0.2708 | 0.2189 | 0 | 0 | 0.1275 | 0.047 | 0.1761 | 0.1796 | 0.2917 |
| MSC | 0.2571 | 0.0253 | 0.0911 | 0.0327 | 0 | 0.0872 | 0.1949 | 0.0285 | 0.1121 | 0 | 0 | 0.1217 | 0.1745 | 0 |
| DC | 0 | 0.0998 | 0 | 0.065 | 0.0098 | 0.0119 | 0.0158 | 0.0057 | 0 | 0.0312 | 0 | 0.003 | 0 | 0.0882 |
| pro B-cells | 0 | 0 | 0 | 0 | 0 | 0.0164 | 0.0519 | 0.0924 | 0.0574 | 0.0979 | 0.0048 | 0 | 0 | 0 |
| Macrophages M1 | 0.0114 | 0.0324 | 0.008 | 0 | 0.0226 | 0.0132 | 0 | 0.0371 | 0 | 0 | 0.0032 | 0.001 | 0 | 0 |
| CD4+ Tcm | 0 | 0.0113 | 0.0034 | 0.0156 | 0 | 0.0292 | 0 | 0.0271 | 0.064 | 0 | 0 | 0 | 0 | 0 |
| CD4+ T-cells | 0.1053 | 0.0869 | 0 | 0.1029 | 0.009 | 0 | 0.029 | 0.0623 | 0 | 0 | 0 | 0.0091 | 0.0226 | 0 |
| MEP | 0 | 0 | 0 | 0.0035 | 0.0979 | 0 | 0 | 0 | 0 | 0 | 0.0179 | 0.0473 | 0 | 0 |
| Tgd cells | 0.0309 | 0.0611 | 0 | 0.0448 | 0.0008 | 0.058 | 0.0206 | 0.0152 | 0.0135 | 0 | 0 | 0 | 0 | 0 |
| cDC | 0 | 0 | 0 | 0 | 0 | 0 | 0 | 0 | 0 | 0 | 0 | 0 | 0 | 0 |
| CD4+ Tem | 0.0414 | 0 | 0 | 0.026 | 0.0035 | 0 | 0.0561 | 0.0513 | 0.0107 | 0 | 0 | 0 | 0 | 0 |
| MPP | 0.1359 | 0.2168 | 0.2505 | 0.1009 | 0.0293 | 0.2442 | 0.3395 | 0.148 | 0.1836 | 0.2298 | 0.2003 | 0.035 | 0.0673 | 0.2472 |
| Smooth muscle | 0.0107 | 0.0186 | 0 | 0.0835 | 0.0186 | 0 | 0 | 0 | 0 | 0 | 0 | 0.1029 | 0 | 0 |
| CMP | 0.0213 | 0.024 | 0 | 0.0071 | 0 | 0 | 0 | 0.0119 | 0 | 0 | 0.0035 | 0.0515 | 0.0131 | 0.0864 |
| Macrophages | 0.0854 | 0.0486 | 0.0646 | 0 | 0.0105 | 0 | 0 | 0.0796 | 0 | 0 | 0.084 | 0.0487 | 0 | 0.0777 |
| Preadipocytes | 0.0209 | 0.0486 | 0.0186 | 0.062 | 0.0642 | 0.0246 | 0.0221 | 0.0447 | 0 | 0.0497 | 0.1008 | 0.0333 | 0.0983 | 0.0502 |
| Plasma cells | 0 | 0.0454 | 0 | 0.0337 | 0.0407 | 0.0382 | 0 | 0.0487 | 0 | 0 | 0.0814 | 0 | 0 | 0 |
| CD8+ Tcm | 0.0659 | 0.0979 | 0 | 0.0121 | 0.0083 | 0.0108 | 0 | 0.0552 | 0.0088 | 0.0976 | 0.0648 | 0.0167 | 0.0202 | 0.1494 |
| B-cells | 0 | 0.0361 | 0 | 0 | 0 | 0 | 0 | 0.0535 | 0 | 0 | 0 | 0 | 0 | 0 |
| Memory B-cells | 0 | 0 | 0 | 0 | 0 | 0 | 0 | 0 | 0 | 0 | 0 | 0 | 0 | 0 |
| Class-switched  memory B-cells | 0 | 0 | 0 | 0 | 0.0305 | 0 | 0 | 0.1672 | 0 | 0 | 0 | 0 | 0 | 0 |
| CD8+ Tem | 0.0502 | 0.0147 | 0 | 0.0345 | 0.0104 | 0.0591 | 0 | 0 | 0.0003 | 0 | 0 | 0 | 0 | 0.0462 |
| Tregs | 0 | 0 | 0.043 | 0 | 0 | 0 | 0.2688 | 0 | 0 | 0 | 0.0079 | 0.0102 | 0.005 | 0 |
| Platelets | 0.0692 | 0.1469 | 0.3046 | 0.1377 | 0.2121 | 0.0579 | 0.0324 | 0 | 0.1776 | 0.0605 | 0.0117 | 0.0309 | 0.2388 | 0.0022 |
| CD8+ T-cells | 0.1192 | 0.0823 | 0.0613 | 0.0767 | 0.0066 | 0.164 | 0.0806 | 0.1665 | 0.0105 | 0.0611 | 0.1167 | 0.1191 | 0.0405 | 0.1028 |

| Cell type | 15 | 16 | 17 | 18 | 19 | 20 | 21 | 22 | 23 | 24 | 25 | 26 | 27 | 28 |
| --- | --- | --- | --- | --- | --- | --- | --- | --- | --- | --- | --- | --- | --- | --- |
| NK cells | 0 | 0 | 0 | 0.1424 | 0 | 0 | 0 | 0 | 0 | 0 | 0.0327 | 0.0107 | 0 | 0 |
| Megakaryocytes | 0.0739 | 0.1084 | 0 | 0.0476 | 0.0443 | 0.0663 | 0.1184 | 0.057 | 0 | 0.0882 | 0 | 0.0465 | 0.0207 | 0.0604 |
| Basophils | 0.0611 | 0.2761 | 0.0625 | 0.194 | 0.0509 | 0.1891 | 0.0827 | 0 | 0.3203 | 0.0191 | 0.0618 | 0.1392 | 0 | 0.0768 |
| Neutrophils | 0.0193 | 0.0493 | 0 | 0 | 0.0142 | 0 | 0 | 0.0343 | 0.0234 | 0.0011 | 0.0253 | 0 | 0 | 0.023 |
| Endothelial cells | 0.0626 | 0.0754 | 0.0405 | 0.0267 | 0.0559 | 0.0232 | 0.0879 | 0.02 | 0.012 | 0.0316 | 0 | 0.0231 | 0.1168 | 0.0225 |
| mv Endothelial cells | 0.0394 | 0.0088 | 0.0329 | 0.0128 | 0.0443 | 0.0339 | 0.062 | 0.0363 | 0.0599 | 0.0235 | 0 | 0.0448 | 0.0571 | 0.0243 |
| CLP | 0.0346 | 0.0564 | 0.0691 | 0.1681 | 0.0513 | 0.2162 | 0.081 | 0.0258 | 0.0564 | 0.0766 | 0.0222 | 0.0968 | 0.0744 | 0.0757 |
| Hepatocytes | 0.0606 | 0.0429 | 0.0249 | 0.0132 | 0.0046 | 0 | 0.0275 | 0.0385 | 0 | 0.0429 | 0.0237 | 0.0314 | 0.0101 | 0 |
| ly Endothelial cells | 0.1191 | 0.0741 | 0.0343 | 0 | 0.0844 | 0.0197 | 0.0882 | 0.0343 | 0.0055 | 0.0015 | 0 | 0.0026 | 0.1155 | 0 |
| Melanocytes | 0.022 | 0.0376 | 0.0646 | 0.0164 | 0.0192 | 0.0247 | 0.0442 | 0.0469 | 0.0226 | 0.0257 | 0 | 0.0348 | 0.0428 | 0.0299 |
| naive B-cells | 0 | 0 | 0 | 0 | 0 | 0 | 0 | 0 | 0 | 0.0532 | 0 | 0.0881 | 0 | 0 |
| Myocytes | 0.0437 | 0.1183 | 0.0314 | 0.0324 | 0.026 | 0 | 0.0497 | 0.0517 | 0 | 0.0851 | 0 | 0.0191 | 0 | 0.0485 |
| Th1 cells | 0.0405 | 0.0842 | 0.1051 | 0.0811 | 0.0714 | 0.1703 | 0.1229 | 0.0973 | 0.1169 | 0 | 0.1307 | 0.0186 | 0.1064 | 0.0755 |
| Chondrocytes | 0.1476 | 0.0573 | 0.119 | 0.0962 | 0.0784 | 0.0161 | 0.1451 | 0.1265 | 0.1975 | 0.1396 | 0.0762 | 0.0539 | 0.154 | 0.196 |
| Neurons | 0.0165 | 0.0136 | 0.0196 | 0.0131 | 0.0166 | 0.0268 | 0.0785 | 0.01 | 0.0043 | 0.0222 | 0.0138 | 0.0193 | 0.0048 | 0.0182 |
| NKT | 0 | 0.1417 | 0.2367 | 0 | 0.0211 | 0 | 0.1742 | 0.1335 | 0.2936 | 0.3557 | 0.0044 | 0.2697 | 0.2939 | 0.1707 |
| Monocytes | 0.0111 | 0.103 | 0.0119 | 0.0045 | 0.0235 | 0.0023 | 0 | 0.0651 | 0.0493 | 0.0779 | 0.0524 | 0.0471 | 0 | 0.066 |
| Epithelial cells | 0.1005 | 0.0851 | 0.0852 | 0.0903 | 0.1006 | 0.0394 | 0.0705 | 0 | 0.1156 | 0.0461 | 0.0814 | 0.0653 | 0.0958 | 0.0784 |
| HSC | 0.3045 | 0.226 | 0.2753 | 0.273 | 0.2726 | 0.1797 | 0.3393 | 0.1262 | 0.1923 | 0.3441 | 0.2552 | 0.2998 | 0.0799 | 0.2845 |
| StromaScore | 0.148 | 0.084 | 0.0786 | 0.0564 | 0.0494 | 0.0274 | 0.1312 | 0.107 | 0.0418 | 0.0647 | 0.0464 | 0.0549 | 0.0934 | 0.1505 |
| aDC | 0.0885 | 0.0268 | 0.1139 | 0.1275 | 0 | 0.1433 | 0.037 | 0.1179 | 0.0957 | 0.1266 | 0.0982 | 0.1771 | 0.0905 | 0.0348 |
| Mesangial cells | 0.0302 | 0.0191 | 0.0786 | 0.0174 | 0 | 0.0503 | 0.0418 | 0.0323 | 0.0058 | 0.0209 | 0.024 | 0 | 0.0701 | 0.0048 |
| pDC | 0.0074 | 0 | 0.0236 | 0.0506 | 0.0306 | 0.0718 | 0.0126 | 0 | 0 | 0.0468 | 0.0254 | 0.058 | 0.0023 | 0.0357 |
| Skeletal muscle | 0.0188 | 0.018 | 0.0227 | 0.0262 | 0.0385 | 0.0252 | 0.0288 | 0.0204 | 0.0173 | 0.0267 | 0.023 | 0.0155 | 0.0125 | 0.0398 |
| Keratinocytes | 0.0725 | 0.0452 | 0.0696 | 0.0865 | 0.0957 | 0.0561 | 0.0708 | 0.0378 | 0.0559 | 0.0603 | 0.0882 | 0.0587 | 0.0914 | 0.0911 |
| Mast cells | 0.0226 | 0.0261 | 0.0233 | 0.0317 | 0.0353 | 0.0384 | 0.0345 | 0.0156 | 0.042 | 0.0227 | 0.0329 | 0.0234 | 0 | 0.0055 |
| CD4+ naive T-cells | 0.002 | 0.0501 | 0.0116 | 0 | 0.0083 | 0 | 0.0199 | 0.0464 | 0.0798 | 0.0288 | 0.0594 | 0.0034 | 0.069 | 0 |
| Sebocytes | 0.015 | 0.0149 | 0.0147 | 0.0159 | 0.0204 | 0.014 | 0.0176 | 0.0103 | 0.0173 | 0.0158 | 0.0181 | 0.0133 | 0.0153 | 0.0161 |
| Fibroblasts | 0.1699 | 0.0401 | 0.0894 | 0 | 0.0322 | 0 | 0.1273 | 0.125 | 0.0215 | 0.0717 | 0.0219 | 0.0206 | 0.0427 | 0.1735 |
| Adipocytes | 0.0634 | 0.0526 | 0.0273 | 0.0861 | 0.0107 | 0.0317 | 0.0472 | 0.0689 | 0.0502 | 0.0261 | 0.0708 | 0.0661 | 0.0273 | 0.105 |
| Osteoblast | 0.1539 | 0.0545 | 0.1225 | 0.1097 | 0.421 | 0.4411 | 0.2634 | 0.1511 | 0.2399 | 0.0913 | 0.2448 | 0.1745 | 0.2077 | 0.1369 |
| Pericytes | 0.0279 | 0.0199 | 0.0077 | 0 | 0.0091 | 0 | 0.0076 | 0 | 0.0178 | 0 | 0 | 0 | 0 | 0.0215 |
| Macrophages M2 | 0.0599 | 0.1057 | 0.1625 | 0.0918 | 0.09 | 0.1107 | 0.0983 | 0.0664 | 0.0435 | 0.0699 | 0.1133 | 0 | 0 | 0.0532 |
| Th2 cells | 0.0537 | 0.06 | 0.0889 | 0 | 0.0853 | 0.0394 | 0.0658 | 0.0664 | 0 | 0.0995 | 0 | 0.081 | 0.0607 | 0.0564 |
| MicroenvironmentScore | 0.4113 | 0.3251 | 0.2541 | 0.3015 | 0.341 | 0.2272 | 0.2711 | 0.4322 | 0.3628 | 0.3248 | 0.4408 | 0.2744 | 0.3142 | 0.3436 |
| Erythrocytes | 0 | 0.0221 | 0 | 0 | 0 | 0 | 0 | 0 | 0 | 0.1103 | 0 | 0.0086 | 0 | 0 |
| GMP | 0.1135 | 0.0685 | 0.0456 | 0.0112 | 0.1514 | 0.0005 | 0.1111 | 0.1134 | 0.1341 | 0.0504 | 0.1547 | 0.0633 | 0.0437 | 0 |
| CD4+ memory T-cells | 0.1454 | 0.0916 | 0.1775 | 0.151 | 0.1123 | 0.1201 | 0.1703 | 0.1182 | 0 | 0.1143 | 0.0489 | 0.0903 | 0.0151 | 0 |
| Astrocytes | 0 | 0 | 0.0295 | 0 | 0 | 0.0008 | 0 | 0 | 0 | 0 | 0 | 0 | 0.0471 | 0 |
| ImmuneScore | 0.2633 | 0.2411 | 0.1755 | 0.2451 | 0.2916 | 0.1998 | 0.1399 | 0.3253 | 0.321 | 0.2601 | 0.3945 | 0.2195 | 0.2208 | 0.1931 |
| CD8+ naive T-cells | 0.194 | 0.1304 | 0.185 | 0.1478 | 0.1397 | 0.2241 | 0.0858 | 0.0191 | 0.1258 | 0.1078 | 0.1001 | 0.1104 | 0.2599 | 0.2166 |
| iDC | 0.0922 | 0.0644 | 0.0386 | 0 | 0.1322 | 0 | 0.1005 | 0.1319 | 0.0755 | 0.041 | 0.0942 | 0.0086 | 0.072 | 0.0325 |
| Eosinophils | 0.2188 | 0.1257 | 0.0726 | 0 | 0.1971 | 0.042 | 0.1441 | 0.1847 | 0.1956 | 0.0114 | 0.179 | 0 | 0.2926 | 0.0508 |
| MSC | 0.3311 | 0 | 0.1008 | 0 | 0.1669 | 0 | 0 | 0 | 0.013 | 0.0613 | 0.1152 | 0.5319 | 0.0331 | 0 |
| DC | 0.0273 | 0 | 0.0034 | 0.0012 | 0.0116 | 0 | 0.0001 | 0.0634 | 0.028 | 0.0043 | 0.0479 | 0.1399 | 0.0046 | 0.0321 |
| pro B-cells | 0 | 0 | 0.0111 | 0 | 0 | 0.0698 | 0 | 0 | 0 | 0 | 0 | 0 | 0 | 0.0137 |
| Macrophages M1 | 0.0163 | 0 | 0.0113 | 0.0318 | 0 | 0.0158 | 0 | 0.0107 | 0.0046 | 0.0064 | 0 | 0 | 0.0111 | 0 |
| CD4+ Tcm | 0 | 0 | 0 | 0 | 0 | 0 | 0 | 0 | 0 | 0 | 0 | 0 | 0.002 | 0.0168 |
| CD4+ T-cells | 0 | 0.0064 | 0.0368 | 0.0938 | 0.0229 | 0.0976 | 0 | 0.0543 | 0.0456 | 0.0065 | 0.1439 | 0.0181 | 0.034 | 0.0412 |
| MEP | 0.0917 | 0 | 0.1212 | 0.0588 | 0.0879 | 0.0045 | 0.1206 | 0.0369 | 0.0476 | 0 | 0.0906 | 0.0717 | 0.007 | 0.0902 |
| Tgd cells | 0 | 0 | 0.0078 | 0 | 0 | 0.0704 | 0 | 0.0516 | 0 | 0.0167 | 0.0724 | 0 | 0.0425 | 0 |
| cDC | 0 | 0 | 0 | 0 | 0 | 0 | 0 | 0 | 0 | 0 | 0 | 0.0756 | 0 | 0.0629 |
| CD4+ Tem | 0 | 0 | 0.0723 | 0.07 | 0.037 | 0.0028 | 0.0171 | 0 | 0.0346 | 0.0838 | 0.0318 | 0.1595 | 0 | 0.0827 |
| MPP | 0 | 0.0876 | 0.0358 | 0 | 0.0251 | 0.29 | 0 | 0.0937 | 0.1437 | 0.0666 | 0.0546 | 0 | 0.0344 | 0.0082 |
| Smooth muscle | 0 | 0 | 0.0619 | 0.0557 | 0 | 0.0393 | 0 | 0.0851 | 0.0076 | 0.0509 | 0.0816 | 0 | 0.0189 | 0.0261 |
| CMP | 0.0182 | 0.027 | 0.0207 | 0.0582 | 0.0111 | 0.0548 | 0.0102 | 0.0329 | 0.0623 | 0.0624 | 0 | 0 | 0 | 0 |
| Macrophages | 0.0835 | 0.0511 | 0.1152 | 0.0862 | 0.0816 | 0.0675 | 0.0311 | 0.0636 | 0.0729 | 0.0578 | 0.0776 | 0 | 0 | 0.0452 |
| Preadipocytes | 0 | 0.0254 | 0.0041 | 0.045 | 0.0913 | 0.0401 | 0.0321 | 0.0181 | 0.0535 | 0.1647 | 0.0611 | 0.044 | 0 | 0.1264 |
| Plasma cells | 0 | 0 | 0.0492 | 0 | 0.0176 | 0.0344 | 0 | 0 | 0 | 0.079 | 0.0572 | 0 | 0.03 | 0.0599 |
| CD8+ Tcm | 0 | 0 | 0.0242 | 0 | 0.0666 | 0.1451 | 0.022 | 0.072 | 0.0224 | 0.0976 | 0.0058 | 0.0415 | 0 | 0 |
| B-cells | 0 | 0 | 0 | 0 | 0 | 0 | 0 | 0 | 0 | 0.1829 | 0 | 0.0901 | 0 | 0 |
| Memory B-cells | 0 | 0 | 0 | 0 | 0 | 0 | 0 | 0 | 0 | 0.0745 | 0 | 0.0089 | 0 | 0 |
| Class-switched  memory B-cells | 0 | 0 | 0 | 0 | 0 | 0 | 0 | 0.0439 | 0 | 0 | 0 | 0.1188 | 0 | 0 |
| CD8+ Tem | 0 | 0 | 0 | 0.0103 | 0.0055 | 0.0524 | 0 | 0 | 0 | 0.036 | 0 | 0.0011 | 0 | 0 |
| Tregs | 0 | 0 | 0 | 0 | 0 | 0.0119 | 0 | 0 | 0 | 0 | 0 | 0 | 0 | 0 |
| Platelets | 0.1028 | 0.1464 | 0 | 0.0672 | 0.0583 | 0.0917 | 0.1422 | 0.0413 | 0 | 0.1216 | 0 | 0.0871 | 0 | 0.0123 |
| CD8+ T-cells | 0.0718 | 0.0593 | 0.1197 | 0.0175 | 0.1634 | 0.1355 | 0.1515 | 0.0725 | 0.0803 | 0.1369 | 0.1541 | 0.1074 | 0.0766 | 0.1328 |

| Cell type | 29 | 30 | 31 | 32 | 33 | 34 | 35 | 36 | 37 | 38 | 39 | 40 | 41 | 42 |
| --- | --- | --- | --- | --- | --- | --- | --- | --- | --- | --- | --- | --- | --- | --- |
| NK cells | 0.0705 | 0 | 0.093 | 0 | 0 | 0 | 0 | 0 | 0 | 0.1177 | 0 | 0 | 0 | 0 |
| Megakaryocytes | 0.0107 | 0.0883 | 0.0227 | 0.0789 | 0 | 0.0504 | 0 | 0.0795 | 0.1066 | 0 | 0.0774 | 0.0406 | 0.1551 | 0.0963 |
| Basophils | 0.227 | 0.1206 | 0 | 0.1862 | 0.1272 | 0.0324 | 0.1285 | 0.0474 | 0 | 0.0874 | 0.1439 | 0.2183 | 0.2643 | 0.093 |
| Neutrophils | 0.0329 | 0 | 0 | 0.0466 | 0.013 | 0 | 0.0275 | 0 | 0.0577 | 0.0672 | 0 | 0.0366 | 0.0429 | 0 |
| Endothelial cells | 0 | 0.004 | 0.0482 | 0.0484 | 0.0647 | 0 | 0.0122 | 0.0616 | 0.0401 | 0.0823 | 0 | 0.0949 | 0.0241 | 0.0459 |
| mv Endothelial cells | 0 | 0 | 0.1063 | 0.0205 | 0.0217 | 0 | 0 | 0.0499 | 0.0254 | 0.0968 | 0 | 0.1159 | 0.0105 | 0 |
| CLP | 0.0759 | 0 | 0.097 | 0.0917 | 0.106 | 0.1128 | 0.1062 | 0.0776 | 0.0929 | 0.1144 | 0.0795 | 0.0477 | 0.1519 | 0.0212 |
| Hepatocytes | 0.0458 | 0.015 | 0.0036 | 0.0215 | 0.0405 | 0.0177 | 0.0294 | 0.0255 | 0.0301 | 0 | 0.03 | 0.0317 | 0.0409 | 0.0014 |
| ly Endothelial cells | 0.054 | 0 | 0.0252 | 0.0815 | 0.1087 | 0.0096 | 0.0192 | 0.182 | 0.0115 | 0.0991 | 0 | 0.1074 | 0.0467 | 0.0639 |
| Melanocytes | 0.0099 | 0.033 | 0.0204 | 0.0073 | 0.0118 | 0.0281 | 0.0338 | 0.0185 | 0.023 | 0 | 0.0208 | 0.0332 | 0.0245 | 0.0421 |
| naive B-cells | 0 | 0 | 0 | 0 | 0 | 0 | 0 | 0 | 0 | 0 | 0 | 0 | 0 | 0 |
| Myocytes | 0.0471 | 0 | 0.0265 | 0.064 | 0 | 0.0039 | 0.0435 | 0.042 | 0.0178 | 0 | 0.0211 | 0.0338 | 0.0294 | 0.0278 |
| Th1 cells | 0 | 0.023 | 0.0535 | 0.0807 | 0.104 | 0.0221 | 0.1637 | 0 | 0.0863 | 0.1493 | 0.079 | 0.0424 | 0.1166 | 0.011 |
| Chondrocytes | 0.1441 | 0.142 | 0.1479 | 0.1525 | 0.152 | 0.163 | 0.1989 | 0.1364 | 0.1471 | 0.1365 | 0.0666 | 0.1264 | 0.1118 | 0.1132 |
| Neurons | 0.0129 | 0.0139 | 0.0315 | 0.0403 | 0.0217 | 0.0175 | 0.0093 | 0.0485 | 0.0088 | 0.0168 | 0.0166 | 0.0218 | 0.0206 | 0.0155 |
| NKT | 0.1655 | 0.3174 | 0.2078 | 0 | 0.1998 | 0.2203 | 0.1629 | 0.3108 | 0.3921 | 0.1417 | 0.1138 | 0.0095 | 0.2445 | 0.193 |
| Monocytes | 0.1548 | 0 | 0.0123 | 0.0564 | 0.0934 | 0.0289 | 0.0088 | 0.0328 | 0.0122 | 0.0402 | 0.0539 | 0.0946 | 0.0014 | 0.0974 |
| Epithelial cells | 0.0605 | 0.0356 | 0.0384 | 0.0291 | 0.1251 | 0 | 0.038 | 0.0538 | 0.0723 | 0.0802 | 0.1008 | 0.0001 | 0.0415 | 0.0532 |
| HSC | 0 | 0.2995 | 0.1977 | 0.2701 | 0.1389 | 0.2018 | 0.1646 | 0.2139 | 0.3412 | 0.0497 | 0.1092 | 0.062 | 0.2609 | 0.3555 |
| StromaScore | 0.0492 | 0.0806 | 0.1148 | 0.0937 | 0.0972 | 0.116 | 0.0832 | 0.1268 | 0.1562 | 0.0836 | 0.0344 | 0.0976 | 0.0775 | 0.0539 |
| aDC | 0.0873 | 0.1434 | 0.1051 | 0.0593 | 0.058 | 0.1148 | 0 | 0.1424 | 0.0685 | 0.1044 | 0.06 | 0.1253 | 0.0892 | 0.1342 |
| Mesangial cells | 0.1395 | 0.0042 | 0.054 | 0.0487 | 0.0471 | 0.073 | 0 | 0.0466 | 0.0181 | 0.0368 | 0 | 0 | 0.0131 | 0.0791 |
| pDC | 0.2528 | 0.0446 | 0.1563 | 0.0124 | 0.022 | 0 | 0.0171 | 0.0036 | 0.0059 | 0.1873 | 0.0469 | 0.0238 | 0.0244 | 0.0851 |
| Skeletal muscle | 0.039 | 0.028 | 0.0112 | 0.0549 | 0 | 0.032 | 0.0357 | 0.0114 | 0.0212 | 0.0163 | 0.0287 | 0.0217 | 0.0209 | 0.0147 |
| Keratinocytes | 0.0664 | 0.0285 | 0.0444 | 0.0488 | 0.1202 | 0.0048 | 0.0789 | 0.0724 | 0.0584 | 0.0852 | 0.0948 | 0.0464 | 0.0556 | 0.0668 |
| Mast cells | 0.0394 | 0.0202 | 0 | 0.0338 | 0.0144 | 0.0359 | 0.0054 | 0.011 | 0.0048 | 0.0269 | 0.0101 | 0.0338 | 0.0132 | 0.0149 |
| CD4+ naive T-cells | 0.0133 | 0.0072 | 0.0226 | 0.043 | 0.0356 | 0 | 0.0516 | 0.0714 | 0.0046 | 0.035 | 0.0553 | 0.0227 | 0 | 0 |
| Sebocytes | 0.0131 | 0.0145 | 0.0125 | 0.0139 | 0.0195 | 0.0125 | 0.0135 | 0.0176 | 0.0172 | 0.0179 | 0.0166 | 0.0097 | 0.0136 | 0.0165 |
| Fibroblasts | 0.0679 | 0.0998 | 0.1455 | 0.1024 | 0.117 | 0.1897 | 0.0801 | 0.0767 | 0.1796 | 0.0608 | 0 | 0.0778 | 0.1018 | 0.0355 |
| Adipocytes | 0.0306 | 0.0573 | 0.036 | 0.0366 | 0.0128 | 0.0424 | 0.074 | 0.1153 | 0.0926 | 0.0242 | 0.0687 | 0.0224 | 0.0291 | 0.0264 |
| Osteoblast | 0.1227 | 0.263 | 0.267 | 0.1542 | 0.1397 | 0.0999 | 0.0558 | 0.0892 | 0.1693 | 0.2274 | 0.1265 | 0.1445 | 0.1738 | 0.316 |
| Pericytes | 0.0055 | 0.018 | 0.0046 | 0 | 0.0771 | 0 | 0.0029 | 0 | 0 | 0.0355 | 0.0165 | 0.0297 | 0 | 0.0166 |
| Macrophages M2 | 0 | 0.0746 | 0.0675 | 0.0471 | 0.0208 | 0.1944 | 0.0022 | 0 | 0.119 | 0.0302 | 0.0868 | 0.054 | 0 | 0.0835 |
| Th2 cells | 0.031 | 0.0747 | 0.023 | 0.0709 | 0 | 0.1134 | 0.0842 | 0.0037 | 0.0569 | 0 | 0.003 | 0.08 | 0.0658 | 0 |
| MicroenvironmentScore | 0.3248 | 0.4269 | 0.4077 | 0.4972 | 0.2514 | 0.4948 | 0.3663 | 0.433 | 0.445 | 0.4903 | 0.1506 | 0.3181 | 0.1945 | 0.2337 |
| Erythrocytes | 0 | 0.0487 | 0 | 0 | 0 | 0 | 0 | 0 | 0.0711 | 0 | 0.0255 | 0 | 0 | 0.0657 |
| GMP | 0.1147 | 0.1112 | 0.143 | 0.111 | 0.0468 | 0.0822 | 0.0972 | 0.1338 | 0.0464 | 0.0457 | 0 | 0.1485 | 0.0202 | 0.0429 |
| CD4+ memory T-cells | 0.126 | 0.1923 | 0.0794 | 0.1035 | 0.1075 | 0.153 | 0.0557 | 0.221 | 0.111 | 0.1379 | 0.1516 | 0.1537 | 0.1174 | 0.0451 |
| Astrocytes | 0.0917 | 0 | 0 | 0 | 0 | 0.0142 | 0 | 0 | 0 | 0 | 0 | 0 | 0 | 0 |
| ImmuneScore | 0.2755 | 0.3463 | 0.2929 | 0.4035 | 0.1542 | 0.3787 | 0.2832 | 0.3062 | 0.2888 | 0.4067 | 0.1163 | 0.2205 | 0.117 | 0.1798 |
| CD8+ naive T-cells | 0.2106 | 0.2576 | 0.2125 | 0.172 | 0.1327 | 0.2078 | 0.2136 | 0.1308 | 0.1522 | 0.0681 | 0.1772 | 0.1118 | 0.1251 | 0.1848 |
| iDC | 0.0492 | 0.0848 | 0.1215 | 0.1636 | 0.025 | 0.1013 | 0.1228 | 0.1693 | 0.063 | 0.1207 | 0.0229 | 0 | 0.0822 | 0.0614 |
| Eosinophils | 0.1126 | 0.172 | 0.1349 | 0.2454 | 0.1043 | 0.2775 | 0.2764 | 0.2345 | 0.2679 | 0.1874 | 0 | 0.0496 | 0.0652 | 0 |
| MSC | 0 | 0.0418 | 0.0775 | 0.1008 | 0.2491 | 0.0685 | 0.2211 | 0.1668 | 0 | 0.0032 | 0.045 | 0.2627 | 0 | 0.0173 |
| DC | 0.0031 | 0.0484 | 0.0572 | 0.0662 | 0 | 0.0445 | 0 | 0.0956 | 0.0002 | 0.0656 | 0 | 0 | 0 | 0.0296 |
| pro B-cells | 0.0358 | 0 | 0 | 0 | 0 | 0.0486 | 0 | 0.0494 | 0 | 0.035 | 0.0311 | 0.0067 | 0.0068 | 0 |
| Macrophages M1 | 0 | 0.0271 | 0.0191 | 0.0012 | 0 | 0.0098 | 0 | 0.0218 | 0 | 0 | 0 | 0.0277 | 0.0072 | 0 |
| CD4+ Tcm | 0.0526 | 0.0657 | 0 | 0 | 0.027 | 0 | 0.0609 | 0 | 0 | 0.0307 | 0 | 0.0207 | 0.0356 | 0 |
| CD4+ T-cells | 0 | 0.0885 | 0.0155 | 0.1136 | 0 | 0 | 0.1067 | 0.0309 | 0 | 0.105 | 0.0047 | 0.0826 | 0.0254 | 0.0228 |
| MEP | 0 | 0 | 0.0681 | 0.0418 | 0.0142 | 0 | 0 | 0 | 0.0419 | 0 | 0.0011 | 0.039 | 0 | 0.0447 |
| Tgd cells | 0.0807 | 0 | 0.0303 | 0.0142 | 0.0103 | 0.0193 | 0 | 0.0379 | 0 | 0.0352 | 0.0149 | 0.0867 | 0 | 0 |
| cDC | 0 | 0 | 0 | 0 | 0 | 0 | 0 | 0 | 0 | 0 | 0 | 0 | 0 | 0 |
| CD4+ Tem | 0 | 0.1333 | 0.0062 | 0 | 0 | 0.0639 | 0 | 0 | 0.0406 | 0.1012 | 0 | 0 | 0 | 0.0311 |
| MPP | 0.371 | 0 | 0.0146 | 0.0247 | 0.0658 | 0.4388 | 0.0572 | 0.3723 | 0 | 0.3226 | 0 | 0.0925 | 0.214 | 0 |
| Smooth muscle | 0.0032 | 0.1026 | 0.0956 | 0.0126 | 0 | 0 | 0 | 0 | 0 | 0 | 0.021 | 0.0629 | 0 | 0.0459 |
| CMP | 0.0251 | 0.0475 | 0.014 | 0 | 0 | 0.0775 | 0 | 0 | 0.0175 | 0.0289 | 0 | 0.0144 | 0 | 0 |
| Macrophages | 0 | 0.1021 | 0.084 | 0.0432 | 0 | 0.0969 | 0 | 0 | 0.0904 | 0 | 0.0475 | 0.0336 | 0.0274 | 0.0712 |
| Preadipocytes | 0.0082 | 0.0816 | 0 | 0.0673 | 0 | 0.0337 | 0.0156 | 0.0348 | 0.0285 | 0.0207 | 0.0381 | 0 | 0 | 0.0951 |
| Plasma cells | 0.0569 | 0 | 0.0061 | 0 | 0 | 0.0062 | 0 | 0 | 0 | 0.0823 | 0.0109 | 0 | 0 | 0.0717 |
| CD8+ Tcm | 0 | 0.0776 | 0 | 0 | 0 | 0.0827 | 0.0033 | 0.1215 | 0.0121 | 0.0049 | 0.0565 | 0 | 0 | 0 |
| B-cells | 0 | 0.0224 | 0 | 0 | 0 | 0 | 0 | 0 | 0 | 0 | 0 | 0 | 0 | 0 |
| Memory B-cells | 0 | 0 | 0 | 0 | 0 | 0 | 0.0252 | 0 | 0 | 0 | 0 | 0 | 0 | 0 |
| Class-switched  memory B-cells | 0 | 0.0916 | 0 | 0 | 0 | 0 | 0.0758 | 0 | 0 | 0 | 0 | 0 | 0 | 0 |
| CD8+ Tem | 0 | 0 | 0 | 0 | 0 | 0.0838 | 0 | 0.0409 | 0 | 0.0422 | 0.0018 | 0 | 0 | 0 |
| Tregs | 0 | 0.139 | 0 | 0 | 0.0124 | 0 | 0 | 0.0291 | 0 | 0 | 0 | 0 | 0.0043 | 0.0085 |
| Platelets | 0.0152 | 0.1424 | 0.0284 | 0 | 0.0053 | 0.0347 | 0.0696 | 0.0263 | 0.0969 | 0.0255 | 0.1278 | 0.0812 | 0.1526 | 0.0886 |
| CD8+ T-cells | 0.1175 | 0.0308 | 0.07 | 0.1187 | 0.1004 | 0.0901 | 0.0883 | 0.0421 | 0.0843 | 0.1397 | 0.0053 | 0.0665 | 0 | 0.0235 |

| Cell type | 43 | 44 | 45 | 46 | 47 | 48 | 49 | 50 | 51 | 52 | 53 |
| --- | --- | --- | --- | --- | --- | --- | --- | --- | --- | --- | --- |
| NK cells | 0 | 0 | 0 | 0 | 0 | 0 | 0 | 0 | 0 | 0 | 0.0556 |
| Megakaryocytes | 0.0213 | 0.0723 | 0.1151 | 0.037 | 0 | 0.0984 | 0.0596 | 0.0723 | 0.1031 | 0.1451 | 0.1139 |
| Basophils | 0.004 | 0.0592 | 0.1404 | 0.0422 | 0.1664 | 0.2206 | 0.0732 | 0.0164 | 0.3496 | 0.1043 | 0.11 |
| Neutrophils | 0 | 0.0168 | 0.0471 | 0.1108 | 0.0201 | 0.0741 | 0.0055 | 0 | 0.0903 | 0 | 0.0016 |
| Endothelial cells | 0.054 | 0.0675 | 0.0275 | 0.0413 | 0.0889 | 0.0503 | 0.0274 | 0.032 | 0.1035 | 0.1364 | 0.0459 |
| mv Endothelial cells | 0.0544 | 0 | 0.0112 | 0.023 | 0.0705 | 0.0897 | 0 | 0.0822 | 0.1582 | 0.0253 | 0.0143 |
| CLP | 0.0792 | 0.0662 | 0.1194 | 0.0918 | 0.0616 | 0.0615 | 0.0698 | 0.0665 | 0.0506 | 0.0616 | 0.1053 |
| Hepatocytes | 0 | 0.0269 | 0.0238 | 0.0418 | 0.0225 | 0.0248 | 0.0192 | 0.0333 | 0.031 | 0.059 | 0.0359 |
| ly Endothelial cells | 0.0741 | 0.0225 | 0.002 | 0.0084 | 0.0355 | 0.114 | 0 | 0 | 0.1378 | 0.112 | 0.0517 |
| Melanocytes | 0.0119 | 0.054 | 0.0438 | 0.021 | 0.0355 | 0.0387 | 0.0156 | 0.0246 | 0.0232 | 0.0093 | 0.0091 |
| naive B-cells | 0 | 0 | 0 | 0 | 0 | 0 | 0.0099 | 0.0195 | 0 | 0 | 0 |
| Myocytes | 0.0077 | 0.0367 | 0.0376 | 0 | 0.0152 | 0.0292 | 0.0371 | 0.0975 | 0.0445 | 0.0531 | 0.051 |
| Th1 cells | 0.1264 | 0.072 | 0.1176 | 0.1251 | 0.0271 | 0.1027 | 0.0811 | 0.0872 | 0.096 | 0 | 0.0126 |
| Chondrocytes | 0.0329 | 0.1725 | 0.1511 | 0.1531 | 0.1917 | 0.0273 | 0.1879 | 0 | 0.1813 | 0.1505 | 0.1415 |
| Neurons | 0.0061 | 0.0193 | 0.0191 | 0.0101 | 0 | 0.0055 | 0.0245 | 0.0087 | 0.001 | 0.0174 | 0.0129 |
| NKT | 0.1544 | 0.3249 | 0.2741 | 0.1477 | 0.2799 | 0.2093 | 0.169 | 0 | 0.0433 | 0.344 | 0 |
| Monocytes | 0 | 0.0389 | 0.0558 | 0.0377 | 0.0136 | 0.0957 | 0.0341 | 0.1203 | 0.1521 | 0 | 0.0636 |
| Epithelial cells | 0.1178 | 0.0725 | 0.1274 | 0.033 | 0.0409 | 0.0562 | 0.065 | 0.1142 | 0 | 0.0813 | 0.0682 |
| HSC | 0.1008 | 0.3025 | 0.1064 | 0.1627 | 0.0731 | 0.1529 | 0.3114 | 0.144 | 0.0794 | 0.3231 | 0.3459 |
| StromaScore | 0.0636 | 0.0876 | 0.0138 | 0.0275 | 0.0875 | 0.0556 | 0.185 | 0.0174 | 0.1969 | 0.138 | 0.0924 |
| aDC | 0.1303 | 0.0814 | 0.0451 | 0.1036 | 0.1588 | 0.0877 | 0.0792 | 0.1521 | 0.0727 | 0.1022 | 0.0848 |
| Mesangial cells | 0 | 0.0363 | 0.0329 | 0.0065 | 0.0159 | 0.0319 | 0.0519 | 0.055 | 0.0307 | 0.0147 | 0.0289 |
| pDC | 0.0203 | 0.0463 | 0.0514 | 0.0189 | 0.0537 | 0.0272 | 0.0141 | 0.0912 | 0.0021 | 0.0416 | 0.0732 |
| Skeletal muscle | 0.0346 | 0.0246 | 0.01 | 0.0173 | 0.0213 | 0.046 | 0.0377 | 0.0105 | 0.0288 | 0.0175 | 0.0285 |
| Keratinocytes | 0.1092 | 0.0823 | 0.1002 | 0 | 0.0751 | 0.0441 | 0.051 | 0.1253 | 0.0082 | 0.0867 | 0.0831 |
| Mast cells | 0.0158 | 0.0196 | 0.0276 | 0.0188 | 0.0027 | 0.0114 | 0.0015 | 0 | 0.0196 | 0.0121 | 0.04 |
| CD4+ naive T-cells | 0.0325 | 0.033 | 0 | 0 | 0.055 | 0 | 0.0427 | 0 | 0.0568 | 0.0138 | 0 |
| Sebocytes | 0.0171 | 0.0156 | 0.0187 | 0 | 0.0145 | 0.0117 | 0.0108 | 0.0192 | 0.0056 | 0.0171 | 0.0179 |
| Fibroblasts | 0 | 0.0772 | 0 | 0.0137 | 0.0448 | 0.0325 | 0.2282 | 0 | 0.2387 | 0.0802 | 0.095 |
| Adipocytes | 0.0731 | 0.0306 | 0 | 0 | 0.0413 | 0.0283 | 0.1143 | 0.0029 | 0.0516 | 0.0594 | 0.044 |
| Osteoblast | 0.3296 | 0.0909 | 0.2043 | 0.2395 | 0.1196 | 0 | 0.0356 | 0.0525 | 0.1764 | 0.1235 | 0.1731 |
| Pericytes | 0.0219 | 0.0061 | 0 | 0.0419 | 0.0315 | 0 | 0.0404 | 0.0393 | 0 | 0.009 | 0 |
| Macrophages M2 | 0.0688 | 0.0274 | 0.0229 | 0.0223 | 0 | 0.1444 | 0.286 | 0.1015 | 0.1145 | 0.0125 | 0.0679 |
| Th2 cells | 0 | 0.0469 | 0.0736 | 0 | 0.0978 | 0.0756 | 0.0737 | 0.0756 | 0.0812 | 0.0639 | 0.0908 |
| MicroenvironmentScore | 0.254 | 0.2114 | 0.1412 | 0.1914 | 0.2358 | 0.3102 | 0.3538 | 0.3112 | 0.5409 | 0.2071 | 0.4796 |
| Erythrocytes | 0 | 0 | 0 | 0 | 0 | 0 | 0 | 0.0296 | 0 | 0.0037 | 0.0208 |
| GMP | 0 | 0.1687 | 0.0492 | 0.103 | 0.1237 | 0.0037 | 0.025 | 0 | 0 | 0.0392 | 0.1117 |
| CD4+ memory T-cells | 0.169 | 0.1146 | 0.1407 | 0.1139 | 0.194 | 0.0116 | 0.0562 | 0.1658 | 0.1672 | 0.0527 | 0.1615 |
| Astrocytes | 0 | 0.0187 | 0 | 0.0046 | 0 | 0 | 0 | 0 | 0 | 0 | 0 |
| ImmuneScore | 0.1905 | 0.1237 | 0.1275 | 0.1639 | 0.1483 | 0.2546 | 0.1689 | 0.2937 | 0.344 | 0.0691 | 0.3871 |
| CD8+ naive T-cells | 0.3207 | 0.0947 | 0.1679 | 0.2501 | 0.1823 | 0.0592 | 0.1594 | 0.1756 | 0.0264 | 0.1388 | 0 |
| iDC | 0 | 0.0402 | 0.0768 | 0 | 0 | 0.0174 | 0.027 | 0.0536 | 0.0808 | 0.0744 | 0.1608 |
| Eosinophils | 0.071 | 0.0723 | 0.0144 | 0.0391 | 0.0229 | 0.0844 | 0.0442 | 0 | 0.1154 | 0 | 0.2922 |
| MSC | 0.0555 | 0.0632 | 0 | 0 | 0.0388 | 0.0282 | 0.0161 | 0.1597 | 0.0979 | 0 | 0.0409 |
| DC | 0 | 0 | 0 | 0 | 0 | 0 | 0 | 0.1583 | 0.0164 | 0.0544 | 0.068 |
| pro B-cells | 0.0258 | 0.0402 | 0.0805 | 0 | 0.0453 | 0 | 0 | 0.0345 | 0 | 0.0055 | 0 |
| Macrophages M1 | 0.0424 | 0.0126 | 0.0149 | 0.0199 | 0.0118 | 0 | 0 | 0 | 0 | 0.0207 | 0 |
| CD4+ Tcm | 0 | 0 | 0 | 0.0057 | 0 | 0 | 0 | 0.0425 | 0 | 0 | 0.0755 |
| CD4+ T-cells | 0.0505 | 0.0058 | 0 | 0 | 0.0693 | 0 | 0.0368 | 0.0267 | 0.0738 | 0.007 | 0.0281 |
| MEP | 0.1316 | 0 | 0 | 0.0635 | 0 | 0 | 0.1268 | 0.088 | 0 | 0.0379 | 0 |
| Tgd cells | 0.0723 | 0.0054 | 0 | 0.0856 | 0.0288 | 0.0381 | 0 | 0 | 0.0473 | 0 | 0 |
| cDC | 0 | 0 | 0 | 0 | 0 | 0 | 0 | 0.1291 | 0 | 0.0872 | 0 |
| CD4+ Tem | 0 | 0.0215 | 0 | 0 | 0 | 0 | 0.0036 | 0.039 | 0 | 0 | 0.0879 |
| MPP | 0.0101 | 0.0481 | 0.2563 | 0.1002 | 0.0826 | 0.0989 | 0.0066 | 0.0002 | 0.1176 | 0 | 0.0004 |
| Smooth muscle | 0.0494 | 0.0132 | 0.0115 | 0.0441 | 0.0495 | 0.0458 | 0.062 | 0 | 0 | 0 | 0 |
| CMP | 0 | 0 | 0 | 0.0391 | 0 | 0.0417 | 0.0087 | 0.0159 | 0.0135 | 0.0192 | 0.0328 |
| Macrophages | 0.0525 | 0 | 0 | 0.007 | 0 | 0.0722 | 0.1033 | 0.0844 | 0.0484 | 0 | 0.0316 |
| Preadipocytes | 0 | 0.0131 | 0.0557 | 0 | 0 | 0.0046 | 0.0158 | 0.0821 | 0.0066 | 0.0447 | 0.0738 |
| Plasma cells | 0.0277 | 0 | 0.0413 | 0.077 | 0.0129 | 0.0159 | 0.0324 | 0.0042 | 0 | 0.0237 | 0 |
| CD8+ Tcm | 0 | 0.0984 | 0.0123 | 0.0562 | 0.0515 | 0.0638 | 0 | 0 | 0.0435 | 0.0365 | 0 |
| B-cells | 0.0194 | 0 | 0 | 0.0245 | 0 | 0 | 0.0279 | 0.0044 | 0 | 0 | 0 |
| Memory B-cells | 0 | 0 | 0 | 0.0022 | 0 | 0 | 0.0239 | 0 | 0 | 0 | 0 |
| Class-switched  memory B-cells | 0 | 0 | 0 | 0 | 0 | 0 | 0 | 0 | 0 | 0 | 0 |
| CD8+ Tem | 0 | 0 | 0.0469 | 0 | 0 | 0 | 0 | 0 | 0 | 0 | 0.0041 |
| Tregs | 0 | 0.0312 | 0 | 0 | 0 | 0 | 0 | 0 | 0 | 0 | 0 |
| Platelets | 0.0019 | 0.0955 | 0.1874 | 0.1444 | 0 | 0.0861 | 0.0351 | 0.118 | 0.1352 | 0.1442 | 0.1837 |
| CD8+ T-cells | 0.1194 | 0.0192 | 0.0373 | 0 | 0.1379 | 0.1073 | 0.0381 | 0.1673 | 0.0517 | 0.0619 | 0.0078 |

References

1. Yang W, Xing X, Yeung SJ, Wang S, Chen W, Bao Y, Wang F, Feng S, Peng F, Wang X *et al*: **Neoadjuvant programmed cell death 1 blockade combined with chemotherapy for resectable esophageal squamous cell carcinoma**. *J Immunother Cancer* 2022, **10**(1).

2. Liu J, Yang Y, Liu Z, Fu X, Cai X, Li H, Zhu L, Shen Y, Zhang H, Sun Y *et al*: **Multicenter, single-arm, phase II trial of camrelizumab and chemotherapy as neoadjuvant treatment for locally advanced esophageal squamous cell carcinoma**. *J Immunother Cancer* 2022, **10**(3).

3. Luo HY, Lu J, Bai YX, Mao T, Wang J, Fan QX, Zhang YP, Zhao KL, Chen ZD, Gao SG *et al*: **Effect of Camrelizumab vs Placebo Added to Chemotherapy on Survival and Progression-Free Survival in Patients With Advanced or Metastatic Esophageal Squamous Cell Carcinoma: The ESCORT-1st Randomized Clinical Trial**. *JAMA* 2021, **326**(10):916-925.

4. Shen X, Zhao B: **Efficacy of PD-1 or PD-L1 inhibitors and PD-L1 expression status in cancer: meta-analysis**. *BMJ* 2018, **362**:k3529.

5. Rodeghiero F, Stasi R, Gernsheimer T, Michel M, Provan D, Arnold DM, Bussel JB, Cines DB, Chong BH, Cooper N *et al*: **Standardization of terminology, definitions and outcome criteria in immune thrombocytopenic purpura of adults and children: report from an international working group**. *Blood* 2009, **113**(11):2386-2393.

6. Malik A, Sayed AA, Han PP, Tan MMH, Watt E, Constantinescu-Bercu A, Cocker ATH, Khoder A, Saputil RC, Thorley E *et al*: **The role of CD8**

**T-cell clones in immune thrombocytopenia**. *Blood* 2023, **141**(20):2417-2429.

7. Gao Q, Zhu H, Dong L, Shi W, Chen R, Song Z, Huang C, Li J, Dong X, Zhou Y *et al*: **Integrated Proteogenomic Characterization of HBV-Related Hepatocellular Carcinoma**. *Cell* 2019, **179**(5):1240.

8. Feng J, Ding C, Qiu N, Ni X, Zhan D, Liu W, Xia X, Li P, Lu B, Zhao Q *et al*: **Firmiana: towards a one-stop proteomic cloud platform for data processing and analysis**. *Nat Biotechnol* 2017, **35**(5):409-412.

9. Schwanhausser B, Busse D, Li N, Dittmar G, Schuchhardt J, Wolf J, Chen W, Selbach M: **Global quantification of mammalian gene expression control**. *Nature* 2011, **473**(7347):337-342.

10. Ge S, Xia X, Ding C, Zhen B, Zhou Q, Feng J, Yuan J, Chen R, Li Y, Ge Z *et al*: **A proteomic landscape of diffuse-type gastric cancer**. *Nat Commun* 2018, **9**(1):1012.

11. Aran D, Hu Z, Butte AJ: **xCell: digitally portraying the tissue cellular heterogeneity landscape**. *Genome Biol* 2017, **18**(1):220.

12. Varghese F, Bukhari AB, Malhotra R, De A: **IHC Profiler: an open source plugin for the quantitative evaluation and automated scoring of immunohistochemistry images of human tissue samples**. *PLoS One* 2014, **9**(5):e96801.

13. Park MD, Reyes-Torres I, LeBerichel J, Hamon P, LaMarche NM, Hegde S, Belabed M, Troncoso L, Grout JA, Magen A *et al*: **TREM2 macrophages drive NK cell paucity and dysfunction in lung cancer**. *Nat Immunol* 2023.
